# Development and validation of a novel Simoa assay for NPTX2 in Alzheimer's disease and Down syndrome

**DOI:** 10.1002/alz.70241

**Published:** 2025-06-16

**Authors:** Mathias Sauer, Bárbara Fernandes Gomes, Parasto Shahrouki, Juan Lantero‐Rodriguez, Laia Montoliu‐Gaya, Elena Camporesi, Olivia Belbin, Daniel Alcolea, Ashish Kumar, Mitu Sharma, Sangeeta Singh, Gunnar Brinkmalm, Johan Gobom, María Carmona‐Iragui, Michael Schöll, Shorena Janelidze, Gagan Deep, Kaj Blennow, Henrik Zetterberg, Ann Brinkmalm, Alberto Lleó, Juan Fortea, Oskar Hansson, Nicholas J. Ashton, Johanna Nilsson

**Affiliations:** ^1^ Department of Psychiatry and Neurochemistry, Institute of Neuroscience and Physiology the Sahlgrenska Academy at the University of Gothenburg Mölndal Sweden; ^2^ Sant Pau Memory Unit, Hospital de la Santa Creu i Sant Pau – Biomedical Research Institute Sant Pau, Sant Antoni Maria Claret Barcelona Spain; ^3^ Centro de Investigación Biomédica en Red de Enfermedades Neurodegenerativas (CIBERNED), Fuencarral‐El Pardo Madrid Spain; ^4^ Department of Internal Medicine‐Gerontology and Geriatric Medicine Wake Forest University School of Medicine Winston‐Salem North Carolina USA; ^5^ Barcelona Down Medical Center Fundació Catalana Síndrome de Down, Carrer del Comte Borrell Barcelona Spain; ^6^ Wallenberg Centre for Molecular and Translational Medicine University of Gothenburg Gothenburg Sweden; ^7^ Dementia Research Centre, Institute of Neurology University College London Queen Square London UK; ^8^ Department of Psychiatry, Cognition and Aging Psychiatry Sahlgrenska University Hospital Mölndal Sweden; ^9^ Clinical Memory Research Unit, Department of Clinical Sciences Malmö Clinical Neurochemistry Laboratory Lund University Malmö Sweden; ^10^ Sahlgrenska University Hospital Mölndal Sweden; ^11^ Paris Brain Institute, ICM, Pitié‐Salpêtrière Hospital Sorbonne University Paris France; ^12^ Neurodegenerative Disorder Research Center, Division of Life Sciences and Medicine, and Department of Neurology, Institute on Aging and Brain Disorders University of Science and Technology of China and First Affiliated Hospital of USTC Hefei Anhui China; ^13^ Department of Neurodegenerative Disease UCL Institute of Neurology, Queen Square London UK; ^14^ UK Dementia Research Institute at UCL, Unit 3a London UK; ^15^ Hong Kong Center for Neurodegenerative Diseases, Clear Water Bay, Hongkong Science Park China; ^16^ Wisconsin Alzheimer's Disease Research Center University of Wisconsin School of Medicine and Public Health, University of Wisconsin‐Madison Madison Wisconsin USA; ^17^ Memory Clinic, Skåne University Hospital Malmö Sweden; ^18^ Banner Alzheimer's Institute and University of Arizona, Phoenix Tucson Arizona USA; ^19^ Banner Sun Health Research Institute Sun City Arizona USA

**Keywords:** Alzheimer's disease, biomarker, cerebrospinal fluid, down syndrome, neuronal pentraxin 2, Simoa, synaptic biomarker

## Abstract

**INTRODUCTION:**

Synaptic dysfunction and loss are pathological hallmarks of neurodegenerative diseases. Neuronal pentraxin 2 (NPTX2), a presynaptic protein involved in synaptic plasticity, has been linked to cognitive decline in Alzheimer's disease (AD) and other neurodegenerative disorders.

**METHODS:**

We developed and validated a novel single molecule array (Simoa) for NPTX2 in cerebrospinal fluid, which was evaluated in two independent cohorts.

**RESULTS:**

CSF NPTX2 concentration was lower (fold change [FC] 0.82, *p* < 0.01) in AD patients and Down syndrome individuals (FC 0.56, *p* < 0.001), compared with cognitively unimpaired patients (CU). It was also associated with Mini‐Mental State Examination (MMSE) score (*β* = 2.51, *p* < 0.001), tau‐PET (*β* = −0.21, *p* < 0.01), and cortical thickness (*β* = 0.08, *p* < 0.001).

**DISCUSSION:**

We describe the first assay for NPTX2 on the Simoa platform, where we continue to highlight the valuable addition of NPTX2 to routine diagnostics of suspected cognitive impairment in patients as it associates better with cognition than other, more established AD biomarkers.

**Highlights:**

- Novel method validated for measuring CSF NPTX2 on semi‐automated Simoa platform.
- Method validated for use in CSF, where it shows a significant decrease in both AD and DS patients.
- Associated with cognition, neurofibrillary tangles, and cortical thickness in AD patients.
- Associations with cognition are shown to be stronger than those of pTau and NFL.

## 1 BACKGROUND

Synapses are specialized structures within neurons that facilitate the transmission of nerve impulses between adjacent neurons, promoting memory formation and learning.^1^ Synaptic dysfunction and degeneration represent distinctive features of various neurodegenerative conditions, with Alzheimer's disease (AD) being the most prevalent and extensively researched.^2^ In AD, pathological studies suggest a stronger association between synaptic degeneration and cognitive decline compared to amyloid‐β (Aβ) plaque pathology,^3^ the primary defining pathological feature in AD. Therefore, there is a compelling case for incorporating synaptic biomarkers into the routine cerebrospinal fluid (CSF) assessment of cognitive impairment, alongside Aβ_42/40_, phosphorylated tau (p‐tau), and total tau (t‐tau). Such biomarkers could prove valuable for both diagnostic purposes, disease staging, as well as predicting the progression of AD in patients. Moreover, synaptic biomarkers have the potential to serve as effective tools for monitoring the downstream impacts on synaptic function and integrity as a result from treatments in drug trials.

A multitude of studied synaptic proteins, located either in the pre‐synaptic terminal (e.g., synaptotagmin‐1, neuromodulin [GAP‐43], β‐synuclein, synaptosomal‐associated protein 25 [SNAP‐25]) or the post‐synaptic terminal, including neurogranin, have been shown to change their levels significantly in dementia,^2^, ^4^, ^5^, ^6^, ^7^, ^8^ prodromal disease,^6^, ^9^ and in some instances even in preclinical disease.^10^ Changes in biomarker levels of β‐synuclein and SNAP‐25 in AD patients have also been described in blood.^11^, ^12^ It is often observed that GAP‐43, SNAP‐25, β‐synuclein, and neurogranin are not changed in non‐AD dementias.^13^, ^14^, ^15^

In contrast, levels of the synaptic protein neuronal pentraxin 2 (NPTX2) are decreased in the CSF of symptomatic stages in a range of neurodegenerative disorders.^16^ NPTX2 is a secretory glycoprotein made up of 431 amino acids (aa) and bind its ligands in a calcium‐dependent manner.^17^ It is made up of a signal peptide (aa 1–17), a low complexity region (aa 96–109), a coiled motif (aa 129–199), and a pentraxin domain (aa 219–424).^18^ Neuronal pentraxins (NPTXs) regulate synaptic plasticity by recruiting α‐amino‐3‐hydroxy‐5‐methyl‐4‐isoxazolepropionic acid‐type glutamate receptor during exocytosis, where they bind and cluster these receptors together.^19^

NPTXs have gained traction as potential biomarkers for neurodegenerative diseases over the years since they are involved in synaptic plasticity, and are also expressed in regions of the brain affected by AD.^20^, ^21^ Decreased CSF NPTX2 levels in AD patients have been confirmed using either enzyme‐linked immunosorbent assays (ELISA)^22^, ^23^ or mass spectrometry (MS).^24^, ^25^ Decreased levels have also been reported in symptomatic frontotemporal dementia (FTD),^26^, ^27^, ^28^ dementia with Lewy bodies (DLB)^26^, ^27^, ^29^ and atypical parkinsonism (progressive supranuclear palsy [PSP], multiple system atrophy [MSA])^30^ and DS.^31^ MS is commonly used as the gold standard for quantifying biomarkers in different body fluids since it has both high sensitivity, specificity, and robust reproducibility. In recent years, there has been a rise of ultrasensitive automated ELISAs, for example, single molecule array (Simoa). These new platforms offer higher throughput, while still maintaining high analytical sensitivity, which is important when considering biomarkers and other neurodegenerative processes in peripheral fluids.^32^

In this study, we developed and validated a novel Simoa method for the semi‐automated quantification of NPTX2 in < 5 µL of CSF per technical replicate and compared it to an established immunoprecipitation mass spectrometry (IP‐MS) method. We tested its performance in two established cohorts, the BioFINDER pilot cohort, comprising CU individuals and AD patients, and the Down Alzheimer Barcelona Neuroimaging Initiative (DABNI) cohort, comprising CU individuals and DS participants. The cohort consisting of adults with DS was included since DS can cause a genetic form of AD,^33^, ^34^ where by the age of 40, most people with DS have developed both Aβ plaques and tau tangles. DS has a lifetime risk of developing AD of over 95%, which is the main cause of death in this population.^33^, ^35^ In addition to CSF, we also evaluated the performance of this biomarker in plasma and neuron‐derived L1CAM+ small extracellular vesicles (NDE).

**RESEARCH IN CONTEXT**

1. **Systematic review**: Previous studies of the pre‐synaptic protein neuronal pentraxin 2 (NPTX2) have shown promising results in discriminating Alzheimer's disease (AD) patients from cognitively unimpaired patients (CU) in cerebrospinal fluid (CSF). The authors developed a novel digital automated emzyne‐linked immunosorbent assay (ELISA) on the Simoa platform to measure NPTX2 in CSF, both in AD patients and individuals with Down syndrome (DS).
2. **Interpretation**: The authors found that NPTX2 was significantly decreased in both AD patients as well as in individuals with DS. NPTX2 was also significantly associated with Mini‐Mental State Examination (MMSE) score, tau‐positron emission tomography (PET), and cortical thickness. It was also associated with Cambridge Cognitive Examination (CAMCOG) scoring in DS participants with severe intellectual disability.
3. **Future directions**: Our findings suggest that NPTX2 is a promising marker for discriminating AD and DS from CU individuals. Its significant association with the MMSE score also suggests it may be a good candidate for the prediction of cognitive decline, although more longitudinal studies are needed to confirm this.

## 2 MATERIALS AND METHODS

### 2.1 Study design and population

For clinical validation studies, in CSF and plasma, we utilized the BioFINDER pilot cohort (*n* = 99, Table 1), which is a selection of samples from the BioFINDER‐2 study (NCT03174938).^36^ The AD patients all fulfilled the AD criteria stated in the Diagnostic and Statistical Manual and Mental Disorders (fifth edition),^37^ as well as being Aβ‐positive as described previously.^38^ The CU subjects were required to be above the age of 40 and have a Mini‐Mental State Examination (MMSE) score of 27 or above. Furthermore, all CU individuals had negative AD biomarkers (CSF Aβ_1‐42/1‐40_ ratio and phosphorylated tau at Thr181 [p‐tau_181_]). The participants were recruited at Skåne University Hospital between April 2017 and September 2019. Ethical approval was provided by the Regional Ethical Committee in Lund, Sweden. As a second validation cohort, a subset of CU (*n* = 20) and DS (*n* = 200) participants from the DABNI were evaluated. The DS subjects were further divided into asymptomatic AD in Down syndrome (aDS), prodromal AD in Down syndrome (pDS), and dementia AD in Down syndrome (dDS) as previously described.^34^ A summary of the BioFINDER and DABNI cohorts can be found in Table 1. The NDE Discovery cohort included plasma from biochemically defined AD patients (*n* = 24) and age‐matched controls (*n* = 23). The AD patients were clinically assessed for suspected AD and demonstrated no evidence of other neurological conditions (e.g., co‐existing inflammatory or cerebrovascular disease). AD patients exhibited a typical AD CSF biomarkers profile. The control group consisted of patients with minor neurological or psychiatric symptoms, with core CSF biomarker levels within normal ranges. Demographics of the Discovery cohort can be found in Table S1. The use of these patient samples had been approved by the Ethics Committee at the University of Gothenburg (EPN 140811).

**TABLE 1:** Baseline characteristics of the different cohorts

	BioFINDER Pilot			DABNI				
**Group**	**CU** ***n* = 51**	**AD** ***n* = 48**	***p*‐value**	**CU** ***n* = 20**	**aDS** ***n* = 83**	**pDS** ***n* = 23**	**dDS** ***n* = 91**	***p*‐value**
Age (year)	78 (6.6, 66–90)	74 (6.9, 55–87)	*p* < 0.01	48 (12.1, 27–70)	38 (8.7, 18–52)	51 (4.7, 39–62)	51 (5.2, 39–63)	*p* < 0.001
Sex			*p* = 0.04					
Female	30 (59%)	18 (38%)		12 (60%)	28 (34%)	9 (39%)	40 (44%)	
Male	21 (41%)	30 (62%)		8 (40%)	55 (66%)	14 (61%)	51 (56%)	
MMSE	29 (1.4, 25–30)	21 (5.9, 5–30)	*p* < 0.001	29 (0.7, 28–30)				
CSF Aβ1‐42/1‐40	1.05 (0.13, 0.95–1.13)^a^	0.50 (0.10, 0.43–0.56)^a^	*p* < 0.001	0.10 (0.01, 0.098–0.109)^b^	0.081 (0.02, 0.065–0.094)^b^	0.05 (0.01, 0.043–0.059)^b^	0.045 (0.01, 0.04–0.053)^b^	*p* < 0.001
CSF p‐tau_181_	40.0 (13.3, 32.0–50.5)^a^	77.5 (21.5, 63.5–92.3)^a^	*p* < 0.001	31.6 (7.1, 28.7–39.2)^b^	28.3 (17.2, 16.17–39.1)^b^	73.2 (54.9, 37.54–127.3)^b^	121.3 (90.4, 73.90–209.7)^b^	*p* < 0.001
CSF t‐tau	296 (99, 230–357)^a^	559 (199, 474–744)^a^	*p* < 0.001	235 (62, 200–277)^b^	275 (165, 163–360)^b^	540 (357, 350–855)^b^	780 (456, 530–1244)^b^	*p* < 0.001
CSF SNAP‐25	121 (37.2, 95–144)	148 (40.1, 119–171)	*p* = 0.003					
CSF NFL	1120 (430, 845–1460)^a^	1530 (845, 966–2280)^a^	*p* = 0.003	313 (95, 266–400)^b^	366 (233, 225–534)^b^	734 (403, 555–1044)^b^	1094 (631, 705–1625)^b^	*p* < 0.001
Amyloid PET, Centiloids	0.93 (0.05, 0.91–0.98)	1.58 (0.34, 1.35–1.81)	*p* < 0.001					
Tau PET, SUVR	1.18 (0.08, 1.1–1.21)	1.95 (0.69, 1.53–2.53)	*p* < 0.001					
Cortical Thickness, mm	2.69 (0.13, 2.37–2.95)	2.45 (0.19, 1.87–2.75)	*p* < 0.001					

*Notes*: Data are presented as mean (standard deviation, min‐max) for age and MMSE. For Aβ_1‐42/1‐40_, p‐tau_181_, t‐tau, NFL, amyloid‐PET, tau‐PET, and cortical thickness, data are presented as median (median absolute deviation, interquartile range).

Abbreviation: Aβ_1‐42/1‐40_, amyloid beta protein ratio 1‐42/1‐40; AD, Alzheimer's disease; aDS, asymptomatic AD in Down syndrome; CSF, cerebrospinal fluid; CU, cognitively unimpaired; DABNI, Down Alzheimer Barcelona Neuroimaging Initiative; dDS, dementia AD in Down syndrome; MMSE, Mini‐Mental State Examination Score; NFL, neurofilament light; PET, positron emission tomography; pDS, prodromal AD in Down syndrome; P‐tau_181_, phosphorylated tau at amino acid Thr181; SNAP‐25, synaptosomal‐associated protein 25; SUVR, standardized uptake value ratio; T‐tau, total tau.

^a^ Markers measured with a Cobas e601 instrument.

^b^ Markers measured with a Lumipulse G600II instrument.

### 2.2 CSF and plasma analysis

For the BioFINDER pilot cohort, CSF samples were collected by lumbar puncture and centrifuged (2200 × *g* for 10 min at 20°C) in polypropylene tubes before being stored at −80°C pending analysis. The core biomarkers for CSF, Aβ_1‐42/1‐40_ ratio, p‐tau_181_, t‐tau, and neurofilament light (NFL), were analyzed on a Cobas e601 instrument, using the NeuroToolKit (Roche Diagnostics). For the matched plasma samples, whole blood was collected in ethylenediaminetetraacetic acid (EDTA) ‐treated tubes and then subsequently centrifuged (4000 × *g* for 10 min). The supernatant was removed and stored in 1 mL aliquots at −80°C before analysis. Samples used for validation were treated with the same procedures.

For the DABNI cohort, CSF samples were collected following international recommendations previously described.^39^, ^40^ Samples were stored at −80°C without being thawed prior to analysis. Commercially available immunoassays for the LUMIPULSE G600II platform were used to determine levels of CSF Aβ42, Aβ40, t‐tau, and p‐tau_181_ (Lumipulse G assays β‐Amyloid 1‐40 and 1‐42, t‐tau, p‐tau181 from Fujirebio, Ghent, Belgium). All the samples were randomized and blinded before analysis.

The Discovery cohort, consisting of biologically confirmed AD (*n* = 24) and CU patients (*n* = 23) (Table S1), was used for the exosome analysis. All individuals were clinically evaluated at Sahlgrenska University Hospital in Gothenburg, Sweden. AD patients were referred for assessment due to suspected AD and underwent lumbar puncture for the measurement of key CSF biomarkers. Those diagnosed with AD displayed a characteristic AD CSF biomarker profile (CSF Aβ42 < 530 ng/L, p‐tau181 > 60 ng/L, t‐tau > 350 ng/L, measured by INNOTEST ELISA). The CU patients included patients with cognitive complaints but no abnormal CSF biomarker levels.

### 2.3 Small extracellular vesicles (sEV) analysis

#### 2.3.1 Isolation of total sEV (TE) from serum and NDE isolation from TE

TE were isolated from serum of all participants from the Discovery cohort, using a precipitation method described by us previously.^41^, ^42^ Briefly, serum samples were centrifuged sequentially at 500 × *g* for 5 min, 2000 × *g* for 10 min, and 10,000 × *g* for 30 min at 4°C to remove any cell debris and large‐size vesicles. Finally, TE were isolated using ExoQuick (System Biosciences, Palo Alto, California, USA) following the manufacturer's recommendations. TE pellet was dissolved in filtered Dulbecco's phosphate buffered saline (DPBS). For the isolation of L1CAM+ sEV sub‐population, 750 µg of TE were incubated, overnight at 4°C with continuous mixing, with 5 µg of biotin‐labeled L1CAM antibody (ThermoFisher Scientific, Cat. No. 13‐1919‐82). Further, 60 µL of streptavidin‐tagged agarose resin (ThermoFisher, MA, USA) was added for 2 h incubation at room temperature with continuous mixing. sEV bound to agarose resins (L1CAM+ sEV) were centrifuged, and the supernatant containing unbound sEV were removed. Finally, L1CAM+ sEV (NDE) were eluted from beads by adding immunoglobulin G (IgG) elution buffer (ThermoFisher, MA, USA), and pH of the eluate was neutralized by 1 M Tris base (pH = 9). A part of the eluted NDE was lysed with 10X RIPA buffer for further analysis.

### 2.4 Nanoparticle tracking analyses (NTA)

The size and concentration of NDE were analyzed by nanoparticle tracking analysis using Nanosight NS300 (Malvern Instruments, UK), as reported previously.^42^, ^43^, ^44^

### 2.5 Preparation of human brain extract for Western blot analysis and IP‐MS

Frozen *post mortem* tissue from human brain donors was provided by the Netherlands Brain Bank (NBB), Amsterdam, Netherlands. The neuropathological assessment confirming AD and control cases was carried out according to the relative diagnostic criteria and the brain bank's program (https://www.brainbank.nl/brain‐tissue/diagnostics/#item‐1). The superior temporal gyrus was sampled from controls (*n* = 3) and AD (*n* = 3) patients. Approximately 100 mg (±20 mg) of brain tissue was manually cut and homogenized in 1 mL Tris buffered saline (TBS) buffer (20 mM Tris‐HCl, 137 mM NaCl, pH = 7.6, with Complete Protease inhibitor Cocktail, Roche Diagnostic GmbH) for 2 min at 200 Hz using the Tissue Lyser II (Qiagen). After homogenization, samples were centrifuged for 1 h at 31,000 × *g* at +4°C, and the supernatant (representing the soluble fraction) was removed and stored frozen at −80°C pending further analysis. All the steps were performed on ice. The protein concentration of the brain extracts was determined using the DC Protein Assay kit (Bio‐Rad). The general demographics of the brain tissues used are summarized in Table S2. Human brain tissues were used in accordance with the Helsinki declaration and the regional ethics committees at NBB and the University of Gothenburg.

### 2.6 NPTX2 Simoa

NPTX2 was measured in CSF (5 µL), plasma (65 µL), and NDE (30 µL) on a HD‐X instrument (Quanterix) with an in‐house developed 2‐step method. This method employed a rabbit monoclonal capture antibody (EPR15618, Abcam) and biotinylated rabbit monoclonal detector antibody (EPR24020‐38, Abcam). Full‐length recombinant NPTX2 (7816‐NP‐050, Biotechne) was used as the calibrator. All samples, as well as calibrators, were diluted with assay diluent (Tau 2.0, #101556 Quanterix) and ran as singlicates. Internal quality controls were run at the beginning and end of each analytical run to assess for variability, both within and across plates. Assay validation included dilution linearity, spike recovery, parallelism, and between‐run stability (freeze‐thaw stability) as previously described.^45^ The development of this assay was performed at the University of Gothenburg.

### 2.7 NPTX2 Western blot and gel electrophoresis

Samples were loaded onto sodium dodecyl sulfate‐polyacrylamide gel electrophoresis (SDS‐PAGE) gels (12% BisTris), which were run at 120 V for 90 min, with MES buffer. They were equilibrated for 20 min in 1x transfer buffer containing 20% MeOH (gels and buffers were all from BioRad or Life Technologies). After equilibration, the gel was transferred to a nitrocellulose membrane (Protran 0.2 µm, Amersham #10600001) via a semi‐dry blot apparatus for 60 min at room temperature set to 12 mA. Following transfer, the membrane was blocked with 5% low‐fat milk (BioRad #170‐6404) in PBS with 0.05% Tween 20 (PBST). The membrane was subsequently incubated with the primary antibody (EPR15618, Abcam, 1:600) diluted in 5% low‐fat milk with PBST, overnight at +4°C. After incubation, the blots were washed three times with PBST for a total of 30 min, before probing with the secondary IgG‐horseradish peroxidase (HRP) antibody (Cell Signaling #7076, anti‐mouse 1:8000) for 60 min at room temperature. Thereafter, the blot was washed three times for a total of 30 min. Further processing and signal detection by ECL (GE Healthcare #RPN2235) was carried out according to standard procedures using the ChemiDoc XRS+ (BioRad Laboratories). Image analysis and comparison of intensity of the bands were performed with Image J (Fiji). The intensity of the bands was normalized against the intensity of glyceraldehyde‐3‐phosphate dehydrogenase (GAPDH; GAPDH Antibody‐HRP (2D4A7), Novus Biologicals).

### 2.8 NPTX2 mass spectrometry

The NPTX2 MS analysis were carried out, both as a targeted method for measurement of NPTX2 in CSF and plasma, or as an explorative data‐dependent method for the identification of NPTX2 peptides in CSF, plasma, and brain tissues (soluble, TBS)

The data‐dependent explorative part was preceded by an immunoprecipitation, where CSF (250 µL), plasma (500 µL), and brain tissue (soluble, TBS; membrane‐bound, Triton; 50 µL) were immunoprecipitated using Dynabeads M‐280 sheep anti‐mouse IgG (Thermo Fisher Scientific) coupled to the antibodies used for the NPTX2 Simoa assay (EPR15618 and ERP24020‐38). The antibodies were conjugated to the beads at a concentration of 4 µg antibody per 50 µL beads. An automated IP was performed using the KingFisher Flex System (Thermo Fisher Scientific). The samples were incubated with antibody‐coupled beads for 30 min at room temperature, followed by multiple washes with PBS, PBST, PBS with 50 mM ammonium bicarbonate (AMBIC), and elution with 0.5% formic acid. The samples were then analyzed on the Q‐Exactive system, as previously described,^46^ either in their endogenous form, or after being digested using trypsin/Lys C (0.4 µg per sample, Promega Co) overnight at 37°C. In short, samples were reconstituted in 7 µL 8% formic acid/8% acetonitrile in water. 6 µL of the sample was then loaded on an Acclaim PepMap C18 trap column (20 mm, 75 µm internal diameter, 100 Å pore size, Thermo Fisher Scientific, Inc.). The loading buffer for the samples was 0.05% trifluoracetic acid/2% acetonitrile in water. Separation of samples was performed at a flow rate of 300 nL/min, with a 50 min long linear gradient going from 3% to 40% buffer B, where buffer A was 0.1% formic acid in water and buffer B was 0.1% formic acid/84% acetonitrile in water. The mass spectrometer utilized high‐energy collision‐induced dissociation (HCD) for ion fragmentation. For both full scan and MS/MS scans, the resolution was set to 70,000 and the maximum trap injection time to 250 ms.

Sample preparation for the targeted method was performed by an in‐house developed method at the University of Gothenburg, as previously described^47^ and the selection of the NPTX2 peptide measured (VAELEDEK, pos. 177‐184) was based on previous work.^48^ Briefly, the heavy peptide standard was added to 100 µL of CSF or plasma, before the samples were reduced and alkylated to remove cystine disulfides and then subsequently digested with trypsin. An IP (as described above) was performed first for the plasma samples. Quantification of the peptide was performed on a micro‐flow LC‐MS/MS system (6495 Triple Quadrouple LC/MS system, Agilent Technologies), equipped with a Hypersil Gold reversed‐phase column (100 mm, 2.1 mm internal diameter, particle size 1.9, Thermo Fisher Scientific). A detailed description of instrument settings has been previously described.^16^ A validation of the targeted NPTX2 method is described in the Supplementary Material (Figure S1 and Table S3).

### 2.9 Image acquisition and processing

In the BioFINDER cohort, magnetic resonance imaging (MRI) was performed on a Siemens 3T MAGNETOM Prisma scanner (Siemens Medical Solutions). Structural T1‐weighted MRI images were acquired from a magnetization‐prepared rapid gradient echo (MPRAGE) sequence with 1 mm isotropic voxels. PET images were acquired on digital GE Discovery MI scanners. For Aβ‐PET, image acquisition was done 90–110 min post‐injection of ∼185 MBq [^18^F]flutemetamol. For tau‐PET, the acquisition was done 70–90 min post‐injection of ∼370 MBq [^18^F]RO948 and was available for all participants. Image processing was done as described previously. Briefly, PET images were attenuation‐corrected, motion‐corrected, summed, and registered to the closest T1‐weighted MRI processed through the longitudinal pipeline of FreeSurfer version 6.0. Standardized uptake value ratio (SUVR) images were created using the inferior cerebellar grey matter as the reference region for [^18^F]RO948, and the cerebellum for [^18^F]flutemetamol. For Aβ, the region of interest was the average SUVR from a neocortical global region (prefrontal, lateral temporal, parietal, anterior cingulate, and posterior cingulate/precuneus). For tau, a temporal meta‐region‐of‐interest (ROI) comprising of an average bilateral entorhinal, amygdala, fusiform, parahippocampal, inferior, and middle temporal cortex SUVR was used. As a measure of neurodegeneration, the average cortical thickness from an AD‐signature ROI of temporal regions (bilateral entorhinal, inferior and middle temporal, and fusiform cortex) was used.

### 2.10 Data processing and statistical analysis

Statistical analyses were conducted using R (version 4.3.2). The distribution of data was examined for normality by using Shapiro–Wilks test and non‐parametric tests were used for the non‐normally distributed data. Group‐wise differences were determined using two‐sided Mann–Whitney test adjusted for multiple comparisons using Holm correction where applicable. Correlation between continuous variables was assessed using Spearman rank correlations. Associations with clinical features and imaging were addressed using linear regression, data were log‐transformed before, and adjusted for age and sex. The receiver operating characteristics curve (ROC) provided the area under the curve (AUC) value, which was used to evaluate the discriminatory power for the diagnostic performance of the biomarkers. Fold change (FC) was calculated by dividing the NPTX2 concentration by the median NPTX2 concentration of the CU group.

## 3 RESULTS

### 3.1 Validation of the CSF NPTX2 Simoa assay

The lower limit of quantification (LLOQ) and lower limit of detection (LOD) were determined as 2.70 pg/mL and 1.14 pg/mL, respectively (Table S4). In CSF, the assay was performed within acceptable limits for dilution linearity (median recovery 105% [97%–118%]), when diluted between 2‐ to 32‐fold with the custom assay diluent (Figure S2A, Table S5). The assay showed good parallelism with a median recovery rate of 102% (83%–111%, CV = 5%–11.9%; Figure S2B, Table S6), and an acceptable spike recovery with a median recovery rate of 92% (73.5%–134%; Figure S2C, Table S7). To evaluate the effect on NPTX2 of freeze‐thawing CSF a freeze‐thaw stability test was performed (Figure S2D, Table S8). A full description of the validation methods and results is shown in the Supplementary Material.

### 3.2 Validation of the plasma NPTX2 Simoa

To validate the NPTX2 assay for the use in plasma on the Simoa platform, the same experiments were carried out as for the CSF validation. Poor dilution linearity was demonstrated with a median recovery rate of 55% (22%–101%, Figure S3A, Table S9). Moreover, the plasma assay also showed an increase in concentration with higher dilutions, as seen in the parallelism test (Figure S3B, Table S10), and poor spike recovery (Figure S3C, Table S11). The three samples repeatedly frozen and thawed up to six times in the freeze‐thaw test, showed stable concentrations (Figure S3D, Table S12). The last test performed for the plasma validation was a matrix effect test, where a sample with a known concentration was diluted down 2‐fold as far as 128 before it was spiked with the same amount of recombinant protein as in the neat sample (Figure S3E). The average number of enzyme labels per bead (AEB) dropped continuously by approximately half until the 32x dilution, after which the AEB started to increase again.

### 3.3 Correlations between MS and SIMOA assays for NPTX2

**FIGURE 1:**
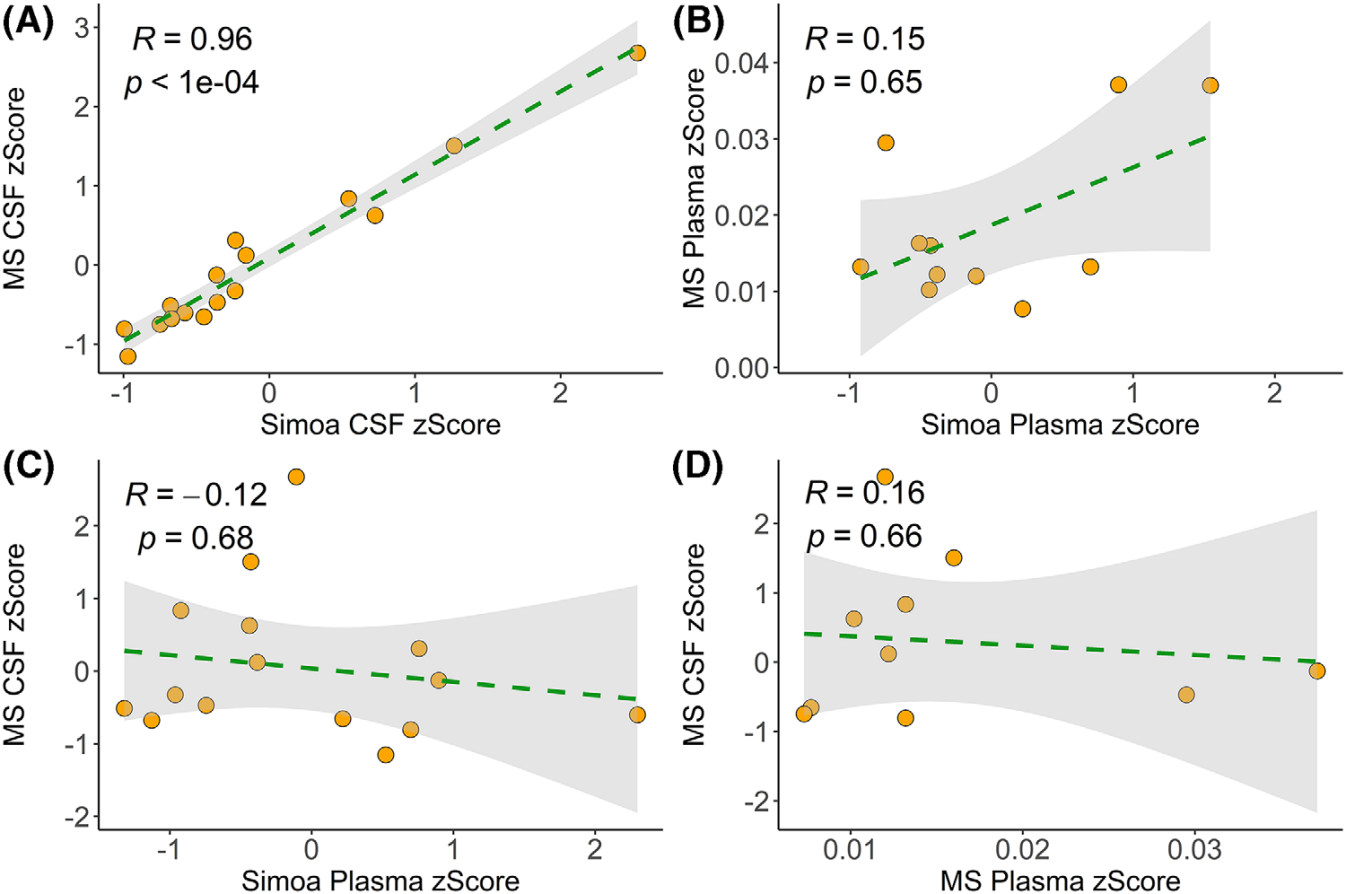
Spearman's rank correlation between neuronal pentraxin 2 (NPTX2) measurements of paired cerebrospinal fluid (CSF) and plasma samples, analyzed with mass spectrometry (MS) and single molecule assay (Simoa). (A) NPTX2 in CSF measured with immunoprecipitation mass spectrometry (IP‐MS) and Simoa. (B) NPTX2 in plasma was measured with IP‐MS and Simoa. (C) NPTX2 was measured in CSF with IP‐MS and in plasma measured with Simoa. (D) NPTX2 was measured in CSF and plasma with IP‐MS

To evaluate whether the Simoa assay was accurately detecting NPTX2, we correlated the Simoa measurements with those from the targeted MS method, which measures the peptide VAELEDEK (aa 177–184). NPTX2 in CSF showed a very strong correlation between both platforms; Spearman's rank correlation coefficient (R) = 0.96, 95% confidence interval (CI_95%_) = 0.89–0.99, *p* < 0.0001 (Figure 1A). However, in plasma, the correlation between the Simoa assay and the IP‐MS assay showed no significant correlation (*R* = 0.16, CI_95% _= −0.49–0.69, *p *= 0.65; Figure 1B). A similar result was obtained when comparing the plasma assay on the Simoa with the CSF MS assay (*R* = −0.12, CI_95% _= −0.59–0.42, *p *= 0.68; Figure 1C) and when comparing NPTX2 in CSF to plasma with the IP‐MS method (*R* = 0.16, CI_95% _= −0.52–0.72, *p *= 0.66; Figure 1D).

### 3.4 Characterization and presence of NPTX2 in CSF, brain, and plasma

**FIGURE 2:**
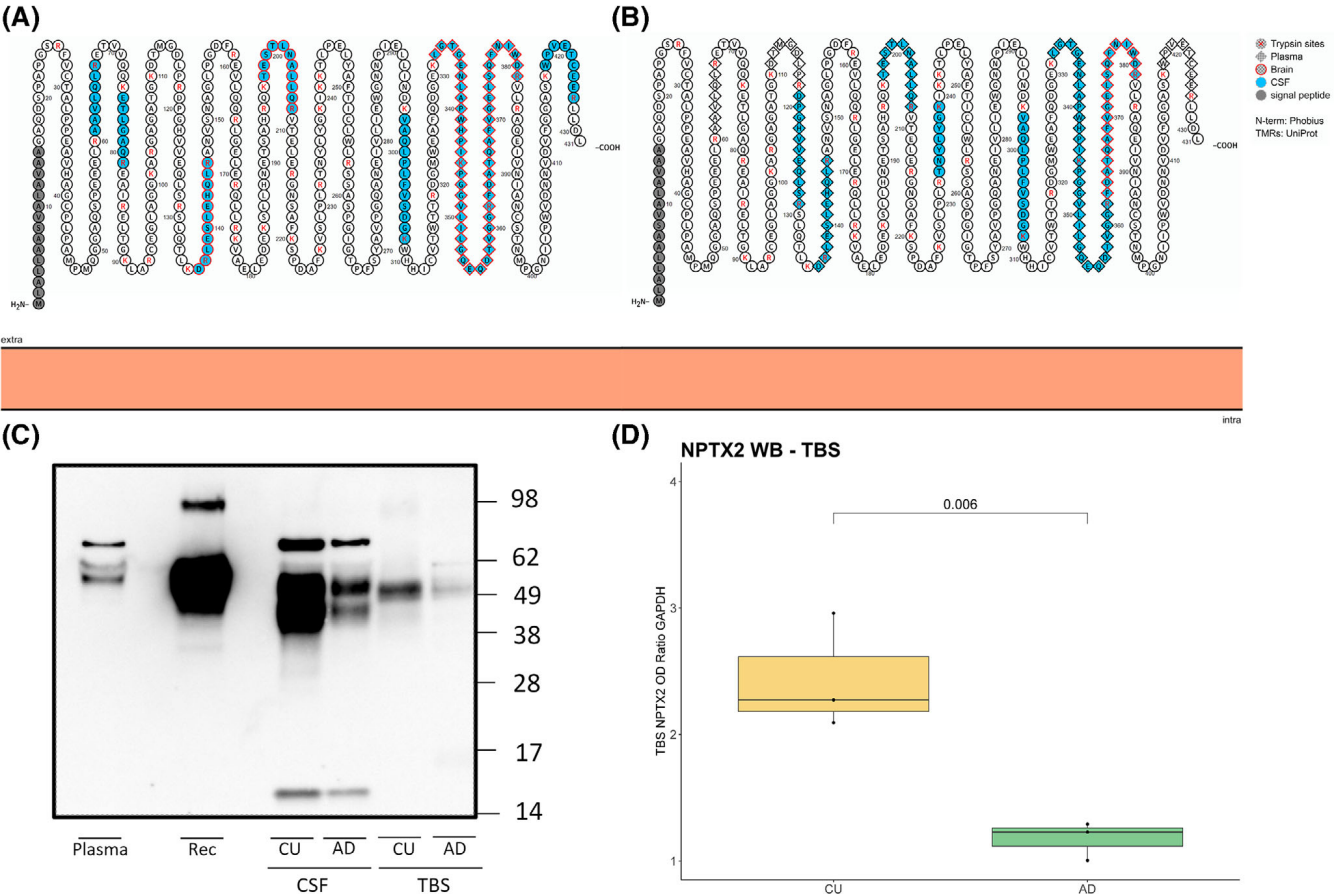
(A–B) Schematic illustration of the neuronal pentraxin 2 (NPTX2) sequence, including the peptides captured by the capture antibody (A, EPR15618, Abcam) and the detection antibody (B, ERP24020‐38, Abcam), where cerebrospinal fluid (CSF) is illustrated by fill color blue, brain by frame color red, plasma by rhombus shape, and trypsin sites are denoted by a red letter. (C) Representative Western blot images of NPTX2 in CSF and brain. cognitively unimpaired (CU), *n* = 3, Alzheimer's disease (AD), *n* = 3. (D) Tris buffered saline (TBS) NPTX2 in CU (*n* = 3) and AD (*n* = 3)

An IP‐MS and a Western blot (WB) were performed to characterize the NPTX2 epitope captured by the assay and to validate the presence of this epitope in CSF, brain, and plasma, respectively (Figure 2). IP‐MS of trypsinized samples were used to test the ability of both capture (Figure 2A) and detector antibody (Figure 2B) capturing NPTX2. For the capture antibody in CSF, fragments were detected throughout the whole protein sequence, whereas for the detector antibody, most fragments captured were located toward the middle or N‐terminal of the protein (Figure 2A,B). On the other hand, the detector antibody had a wider capture range for NPTX2 in plasma, as the capture antibody only detected NPTX2 fragments from plasma in the N‐terminal region. Furthermore, the capture antibody seemed to have a wider range for detecting NPTX2 in brain samples.

For the WB, 4.65 µg/sample protein for CSF, plasma and TBS was run, and the strongest band in all matrices was found at ∼49 kDa (Figure 2C; Figure S4). In CSF and plasma, there is an additional band at ∼62 kDa (Figure S4A,B) and TBS seems to have a multitude of bands ranging from ∼30 to 62 kDa (Figure S4A,C). Overall, AD patients had weaker bands, as seen in both the brain fraction (FC 0.54, CI_95% _= 0.34–0.62, *p* < 0.01; Figure 2C,D) and in CSF (Figure 2C).

### 3.5 CSF NPTX2 in the BioFINDER pilot cohort

**FIGURE 3:**
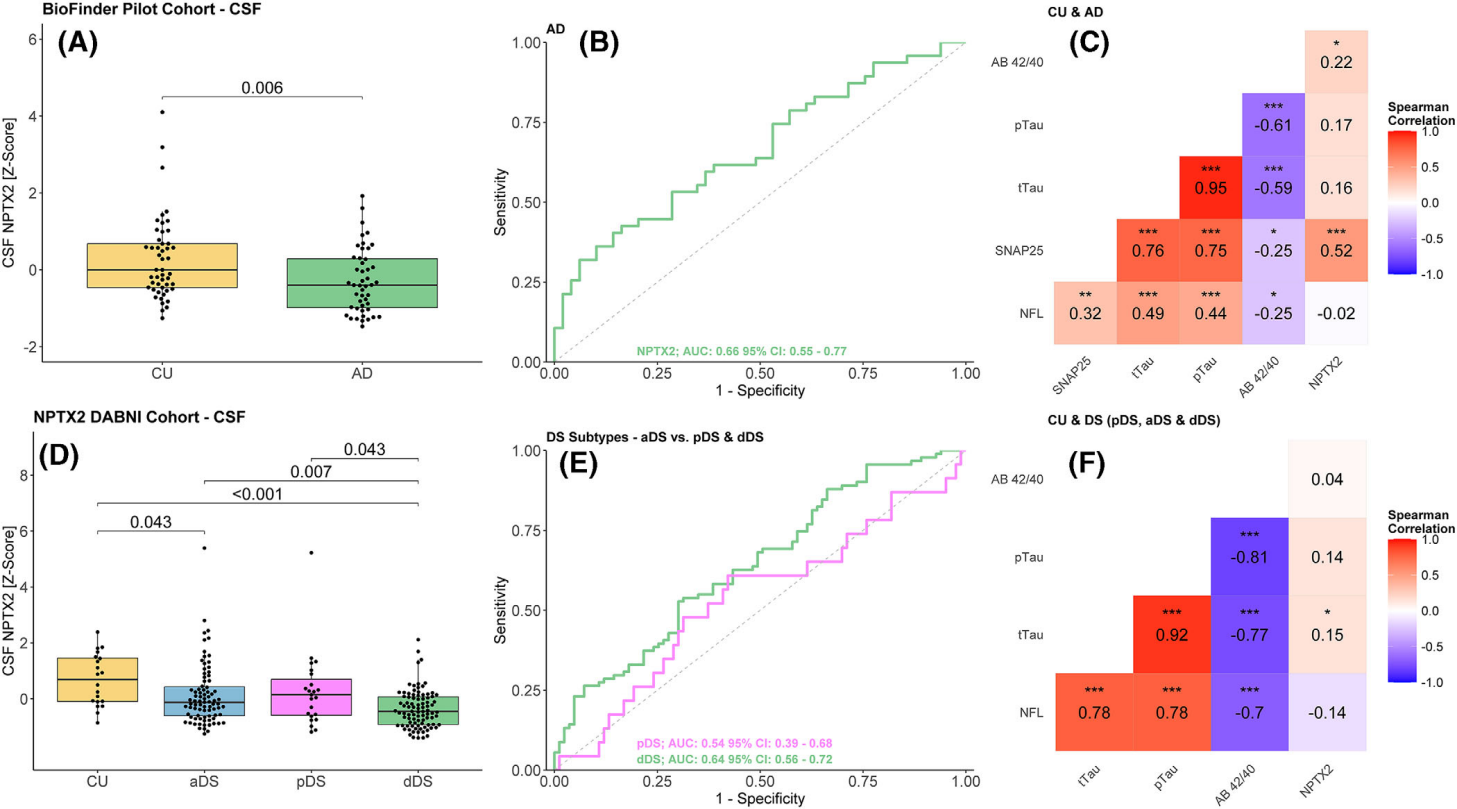
(A–C) BioFINDER Pilot cohort (cerebrospinal fluid [CSF]). (A) Neuronal pentraxin 2 (NPTX2) in cognitively unimpaired (CU) (*n* = 51) and Alzheimer's disease (AD) (*n* = 48) patients. (B) Receiver operating characteristics curve (ROC) curve for NPTX2 in AD compared to CU. (C) Correlations for CSF biomarkers in the full cohort. (D–F) DABNI cohort (CSF). (D) NPTX2 in CU (*n* = 20), prodromal AD in Down syndrome (pDS) (*n* = 23), asymptomatic AD in Down syndrome (aDS) (*n* = 83) and dementia AD in Down syndrome (dDS) (*n* = 91). (E) ROC curve for NPTX2 in the three DS groups compared to CU. (F) Correlations for CSF biomarkers in the full cohort. *p*‐values are indicated by asterisks: *p < 0.05, **p < 0.01, and ***p < 0.001

In the BioFINDER pilot cohort, CSF NPTX2 levels showed a significant decrease in patients with AD, compared to CU (Figure 3A), with a FC in AD of 0.82 (CI_95% _= 0.67–0.99, *p* = 0.006). CSF NPTX2 showed modest accuracy in discriminating AD from CU (AUC = 0.66, CI_95% _= 0.55–0.77; Figure 3B). In the full cohort, CSF NPTX2 had a modest correlation with CSF SNAP‐25 (*R* = 0.52, CI_95%_ = 0.36–0.65, *p* < 0.001) and a weak correlation with CSF Aβ42/40 ratio (*R* = 0.22, CI_95%_ = 0.02–0.41, *p* < 0.05). However, it did not show any significant correlation with the other biomarkers, such as CSF p‐Tau_181_, t‐Tau, and NFL (Figure 3C). In the CU group alone CSF NPTX2 had a good correlation with CSF SNAP‐25 (*R* = 0.71, CI_95%_ = 0.54–0.83, *p* < 0.0001), as well as with both CSF t‐Tau (*R* = 0.62, CI_95%_ = 0.41–0.77, *p* < 0.0001) and with p‐Tau_181_ (*R* = 0.61, CI_95%_ = 0.40–0.76, *p* < 0.0001) (Figure S5B). In the AD group, CSF NPTX2 retained a modest correlation with CSF SNAP‐25 (*R* = 0.59, CI_95%_ = 0.37–0.75, *p* < 0.001) and a weak correlation with CSF t‐Tau (*R* = 0.39, CI_95%_ = 0.11–0.61, *p* < 0.01), CSF p‐Tau_181_ (*R* = 0.46, CI_95%_ = 0.2–0.66, *p* = 0.001), as well as a weak negative correlation with the CSF Aβ_1‐42/1‐40_ ratio (*R* = −0.29, CI_95%_ = −0.53 to −0.01, *p* < 0.05) (Figure S5C).

### 3.6 CSF NPTX2 in the DABNI cohort

In DS individuals, CSF NPTX2 levels showed a significant decrease, compared to CU (Figure S6A). The FC was 0.57 (CI_95% _= 0.43–0.91, *p* < 0.001). For DS subjects, NPTX2 showed moderate accuracy when discriminating between DS and CU (AUC = 0.75, CI_95% _= 0.64–0.85; Figure S6B). When the DS group was stratified into the three subgroups (Figure 3D), the dDS group was most decreased compared to CU (FC = 0.49, CI_95%_ 0.36–0.79, *p* < 0.001) followed by the aDS group (FC = 0.64, CI_95%_ 0.46–0.99, *p* = 0.04). The pDS group was not significantly reduced compared to CU (FC = 0.76, CI_95%_ 0.46–1.23, *p* > 0.05). Furthermore, in discriminating the aDS group from the symptomatic DS groups (pDS and dDS), NPTX2 showed the highest AUC in dDS (AUC = 0.64, CI_95% _= 0.56–0.72; Figure 3E) followed by pDS (AUC = 0.54, CI_95% _= 0.39–0.68; Figure 3E) CSF NPTX2 levels in DS individuals were then assessed in relation to intellectual disability (mild, moderate, severe), both in the full cohort and in the three separate subgroups. No significant differences were found.

In the full cohort, CSF NPTX2 showed only a weak correlation with CSF t‐Tau (*R* = 0.15, CI_95%_ = 0.01–0.28, *p* < 0.05; Figure 3F) and a weak, albeit not significant correlation with CSF p‐Tau_181_ (*R* = 0.14, CI_95%_ = −0.01–0.28, *p* = 0.054) (Figure S5D). In the combined DS group alone, it showed a weak correlation with both CSF p‐Tau_181_ (*R* = 0.18, CI_95%_ = 0.03–0.32, *p* < 0.05) and tTau (*R* = 0.21 CI_95%_ = 0.06–0.35, *p* < 0.01) (Figure S5F). CSF NPTX2 in dDS was also significantly correlated with CSF p‐Tau_181_ (*R* = 0.53, CI_95%_ = 0.35–0.67, *p* < 0.001), CSF t‐Tau (*R* = 0.48, CI_95%_ = 0.29–0.63, *p* < 0.001), as well as the CSF Aβ_1‐42/1‐40_ ratio(*R* = −0.51, CI_95%_ = −0.65 to −0.32, *p* < 0.001) (Figure S5I). There was also a significant correlation between CSF NPTX2 and CSF p‐Tau_181_ (*R* = 0.58, CI_95%_ = 0.40–0.71, *p* < 0.001) and CSF t‐Tau (*R* = 0.51, CI_95%_ = 0.32–0.66, *p* < 0.001) (Figure S5H) in the aDS group.

### 3.7 NPTX2, cognition, brain atrophy, Aβ‐PET, and tau‐PET

**FIGURE 4:**
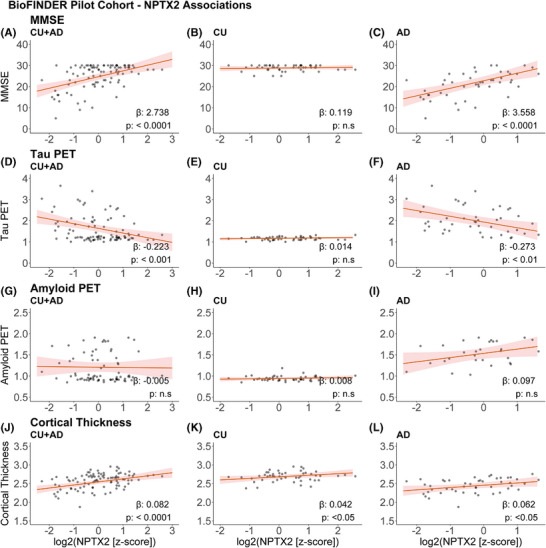
Associations of cerebrospinal fluid (CSF) neuronal pentraxin 2 (NPTX2) in BioFINDER Pilot cohort, adjusted for age and sex. Association with (A–C) Mini‐Mental State Examination (MMSE) score in (A) cognitively unimpaired (CU) + Alzheimer's disease (AD) (*n* = 95), (B) CU (*n* = 49), and (C) AD (*n* = 46); (D–F) tau‐PET in (D) CU + AD (*n* = 95), (E) CU (*n* = 49), (F) AD (*n* = 46); (G–I) amyloid‐PET in (G) CU + AD (*n* = 78), (H) CU (*n* = 49), (I) AD (*n* = 29); (J–L) cortical thickness in (J) CU + AD (*n* = 96), (K) CU (*n* = 49), and (L) AD (*n* = 47)

A significant positive association between CSF NPTX2 levels and MMSE score (β (SE) = 2.74 (0.56), *p *< 0.0001, Figure 4A) was found in the BioFINDER pilot cohort when assessing all patients. In the AD group alone, they were also significantly associated (β (SE) = 3.56 (0.80), *p* < 0.0001, Figure 4C), but not in the CU group (β (SE) = −0.12 (0.23), *p* = 0.60, Figure 4B). CSF NFL and p‐Tau_181_ were significantly associated with MMSE score in the full cohort (Figure S7–8), however, the association is not significant when looking at the AD or CU group alone. There was also a weak but significant association between CSF NPTX2 levels and cortical thickness in the whole cohort (β (SE) = 0.08 (0.02), *p* < 0.0001, Figure 4J), the AD group alone (β (SE) = 0.06 (0.03), *p* < 0.05, Figure 4L) and in the CU group alone (β (SE) = 0.04 (0.02), *p* < 0.05, Figure 4K). A significant negative association was found between tau‐PET measure and CSF NPTX2 levels (β (SE) = −0.22 (0.06), *p* < 0.001, Figure 4D) both in the whole cohort as well as within the AD group alone (β (SE) = −0.27 (0.09), *p* < 0.01, Figure 4F); however, no significant associations were found in the control group (β (SE) = 0.01 (0.01), *p* = 0.27, Figure 4E). Lastly, no significant association was observed with amyloid‐PET measures in any of the groups (Figure 4G–I).

In the DABNI cohort, we investigated the association between NPTX2 and the Cued Recall Test (CRT) in the overall DS group and in the different clinical groups separately (Figure S9). CSF NPTX2 was significantly associated with CRT in the full cohort (β (SE) = 1.25 (0.46), *p* < 0.01, Figure S9A), as well as in the dDS group (β (SE) = 2.12 (0.99), *p* < 0.05, Figure S9D). CSF pTau_181_ was significantly associated with the CRT score in both the full cohort (β (SE) = −1.64 (0.47), *p* < 0.001, Figure S9E) as well as in the aDS group (β (SE) = −0.88 (0.36), *p* < 0.05, Figure S9F). CSF NFL was significantly associated with the CRT score both in the full DS cohort (β (SE) = −1.64 (0.53), *p* < 0.01, Figure S9I), as well as in the dDS group (β (SE) = −2.43 (1.06), *p* < 0.05, Figure S9L).

### 3.8 Plasma NPTX2

NPTX2 in plasma did not show a significant difference between AD and CU; yet a small median increase of NPTX2 in AD was found (FC = 1.11, CI_95%_ = 0.92–1.31, *p *= 0.14; Figure S10A). The plasma concentrations were, on average, 28 times lower than CSF (standard deviation = 14.3). Additionally, there was no correlation between CSF and plasma NPTX2 measured on the Simoa in all patients (*R* = 0.077 *p *= 0.46; Figure S10B) or in the AD group alone (*R* = −0.14 *p* = 0.36; Figure S10D). However, there was a significant moderate correlation between CSF and plasma NPTX2 in the control group (*R* = 0.37 *p* = 0.01; Figure S10C). Regarding the core CSF biomarkers (NFL, Aβ_42/40_, p‐Tau_181_ and t‐Tau), only a weak correlation between plasma NPTX2 and CSF p‐Tau_181_ in the CU group (*R* = 0.31, CI_95%_ = 0.03–0.55, *p* < 0.05; Figure S11C) was found (Figure S11).

Furthermore, no significant association between NPTX2 and MMSE, amyloid‐PET, tau‐PET, or cortical thickness was observed (Figure S12).

### 3.9 NPTX2 in NDE

**FIGURE 5:**
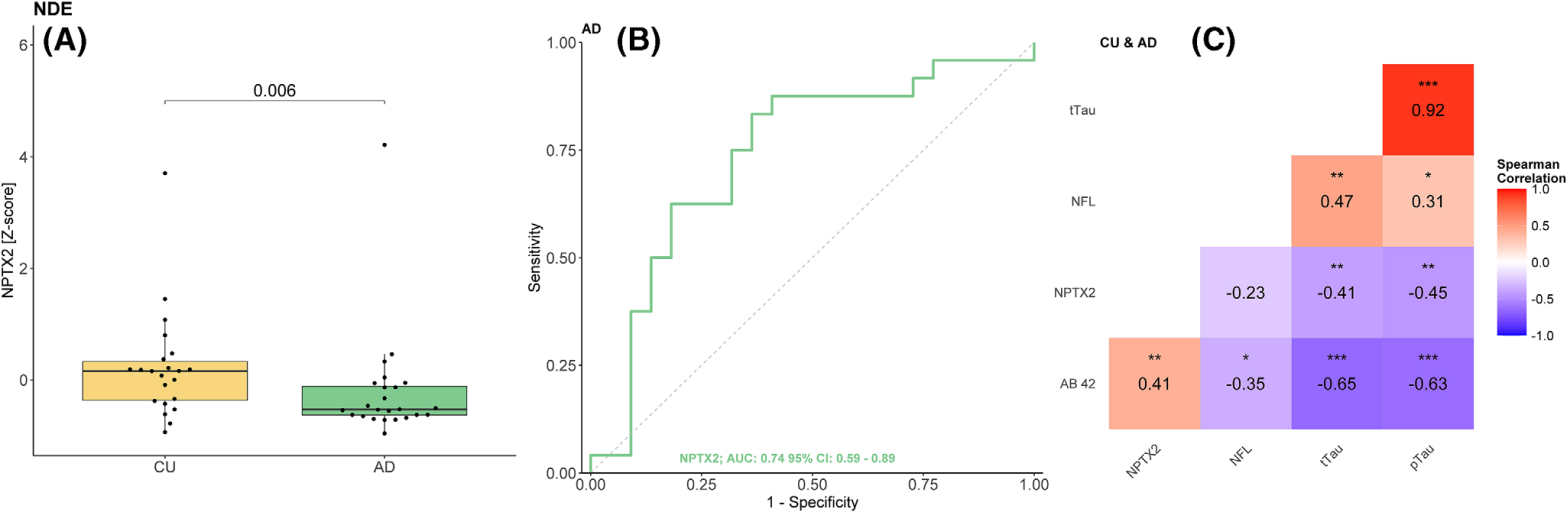
L1CAM+ NDE, (A) neuronal pentraxin 2 (NPTX2) in cognitively unimpaired (CU) (*n* = 23) and Alzheimer's disease (AD) (*n* = 24) patients. (B) Receiver operating characteristics curve (ROC) curve for NPTX2 in AD compared to CU. (C) Correlations for cerebrospinal fluid (CSF) biomarkers and NPTX2 from NDE in all patients. *p*‐values are indicated by asterisks: *p < 0.05, **p < 0.01, and ***p < 0.001

NDE were isolated from plasma and nanoparticle tracking analyses confirmed sEV nature as size of all NDE sample was below 200 nm (mean size for CU = 151.70 ± 3.90 nm, and AD = 148.86 ± 3.140 nm). Interestingly, there was a significant difference between AD and CU (Figure 5A), with a FC in AD of 0.44 (CI_95%_ 0.36–0.80, *p* < 0.01). In the exosomes, NPTX2 showed moderate accuracy in distinguishing AD from controls (AUC = 0.74, CI_95%_ = 0.59–0.89; Figure 5B). Furthermore, NPTX2 in exosomes was negatively correlated with CSF p‐Tau_181_ (*R* = −0.45, CI_95%_ = −0.66 to −0.18, *p* < 0.01; Figure 5C), and CSF t‐Tau (*R* = −0.41, CI_95%_ = −0.63 to −0.14, *p* < 0.01; Figure 3I) in all the patients, and had a significant positive correlation with CSF Aβ_1‐42_ (*R* = 0.41, CI_95%_ = 0.13 to 0.62, *p* < 0.01; Figure 3I). When investigating the AD group and CU separately, no significant correlations were observed (Figure S5H,I).

## 4 DISCUSSION

NPTX2 was first described in 1995,^49^ but it was not characterized in CSF until 2016,^25^ when advances in the MS field had progressed enough to detect and quantify these low‐abundant proteins. Interest in synaptic proteins has risen in the past years, with GAP‐43, SNAP‐25, and neurogranin being among the most studied in AD.^6^, ^9^, ^13^, ^22^ Likewise, there has been an increased focus on NPTX2, which has been highlighted as a prominent biomarker, partly due to its expression in AD‐affected regions in the brain^50^ as well as its involvement in synaptic plasticity^20^, ^21^ and significant changes in multiple neurodegenerative disorders.^16^ Over the past few years, our group has developed an in‐house MS method to measure NPTX2, and demonstrated its application in various disorders.^16^, ^26^, ^30^, ^48^ However, given the prominence of NPTX2 in neurodegenerative disorders, more accessible and automated methods are needed for this biomarker to be applied in routine assessment. In this study, we have developed and validated a Simoa assay to measure NPTX2. We assessed its performance in two independent cohorts, one consisting of AD and healthy controls, in both plasma and CSF, and the other cohort consisting of CSF from DS along the AD continuum and healthy controls. We also assessed it in the context of NDE.

The NPTX2 Simoa assay was first validated according to previously established methods,^45^ and for CSF it performed well within previously set criteria.^45^ There was also a very strong correlation between the Simoa CSF assay and the MS method (*R* = 0.96), implying it has a strong concordance with an established method. Furthermore, peptides from the whole NPTX2 protein were detected when samples were trypsinized and measured with IP‐MS. However, when the same non‐trypsinized samples were analyzed, no endogenous peptides were observed. This suggests that either NPTX2 is present as a full‐length protein in CSF, plasma, and brain, or that the endogenous cleavage sites result in peptides too large for MS quantification. However, the results from the WB, which utilized the same antibodies as the Simoa method, showed the strongest band to be at ∼49 kDa (Figure 2C, Figure S4), further strengthening the notion that it is the full‐length protein being measured. We observed an additional band in all three matrices at ∼62 kDa, which is thought to be a glycosylated form of NPTX2.^17^, ^51^ We also observed an additional band ∼40 kDa in CSF and brain tissue, which could correspond to a smaller cleaved version of NPTX2.

The NPTX2 Simoa assay demonstrated significantly lower NPTX2 levels in AD patients compared to CU patients in CSF, which is in line with a body of literature.^22^, ^23^, ^24^, ^25^, ^26^, ^52^ This significant drop observed in NPTX2 levels in AD patients was also reflected in our WB results (Figure 2C). Furthermore, NPTX2 in CSF was the only biomarker that was associated significantly with MMSE score in AD patients, outperforming previously established biomarkers (e.g., p‐Tau_181_), which serves as evidence of its robustness as a cognitive impairment marker. In our cohort, NFL and p‐Tau_181_ were only significantly associated with MMSE score when combining the CU and the AD groups, but not in these groups alone, which is in line with previous findings.^53^, ^54^, ^55^ The significant drop in NPTX2 levels found in AD patients in our study was observed to a greater extent in participants with DS, especially in the dDS group. This decrease in DS compared to sporadic AD was expected since people with DS have an extra copy of chromosome 21, where the APP gene is located. Mutations in this gene are both necessary and sufficient to cause AD, leading DS to be considered a genetically determined form of AD. By the age of 40, almost all individuals with DS have developed both Aβ plaques, as well as tau tangles, and estimates suggesting that up to 50% will develop AD dementia as they age.^56^, ^57^ Therefore, these results support the validity of the NPTX2 assay. Furthermore, we did not find any association with NPTX2 and intellectual disability. When taken together with the significant association observed with cortical atrophy in AD patients, this suggests that the reduced levels of CSF NPTX2 are not due to neurodevelopmental factors but rather due to synaptic loss and dysfunction in AD patients. Of note, CSF NPTX2 correlated with the CRT scores. The CRT is an adapted test to assess memory in DS individuals, which captures early memory impairments (late 30s to early 40s),^58^, ^59^ has excellent diagnostic performance to diagnose AD dementia,^60^, ^61^ but also has floor effects in dDS.

As mentioned above, the levels of NPTX2 in AD in this study were significantly lower than controls, which is also the case for other neurodegenerative diseases, such as DLB,^26^, ^27^, ^29^ atypical parkinsonism (PSP, MSA)^30^ and FTD.^26^, ^27^, ^28^ This is in contrast to other synaptic proteins measured in CSF, for example, GAP‐43, SNAP‐25, and neurogranin, which are changed only in response to amyloid pathology.^62^ Additionally, NPTX2 has also been associated with neuroinflammatory responses that, in turn, are associated with trauma and neurological diseases,^63^ as well as with blood‐brain barrier dysfunction.^64^ Taken together, these results indicate that NPTX2 is not specific to the underlying pathology of AD, but instead associated with general neurodegeneration. The decrease seen in the various neuronal diseases could be attributed to a reduced neuronal activity, since NPTX2 has been shown to be an immediate early gene, produced as a response to increased synaptic activity.^17^ A further indication of this reduced neuronal activity is the significant association between NPTX2 and MMSE score in AD shown in this study. When compared to other synaptic biomarkers, NPTX2 has shown to be more strongly associated with MMSE score, both in AD and other neuronal degenerative diseases.^22^, ^26^, ^27^, ^30^, ^48^, ^65^ So far, only SNAP‐25 and β‐synuclein have been validated for use on the Simoa platform.^12^, ^66^ However, NPTX2 is a promising synaptic biomarker candidate for neuronal degeneration in a range of neurodegenerative diseases.

The Simoa plasma assay did not correlate with the CSF nor the MS method, where in CSF the NPTX2 levels were decreased in AD compared to CU, whereas the plasma results showed a tendency to be increased in AD compared to controls, which has been previously shown.^67^ One reason for this discrepancy could be the diverse expression of NPTX2 throughout the body. In the brain, NPTX2 is mostly expressed in the anterior pituitary gland and in neurons in the cerebral cortex, but outside the brain, it is also present in the colon, pancreas, prostate gland, testis, vitreous humor, and in endocrine tissues (adrenal gland).^18^, ^50^ Another reason could be the strong matrix effect clearly observed in the plasma assay, where the AEB signal from the Simoa is continuously dropping until the sample has been diluted enough times to remove effects from the plasma matrix (Figure S2E), meaning it needs further optimization. Given that we have shown a small, albeit not significant, change of plasma NPTX2 in AD and moderate correlations with MMSE and tau‐PET this optimization may be warranted. If the problem is the peripherally expressed NPTX2, a circumvent could be to isolate NDE, which is released by neurons in the brain and carry cargo from its source. Due to their inherently small size, they are capable of crossing the blood–brain barrier and are therefore detectable in plasma.^68^ In this study, we have shown, like others,^69^ that the NPTX2 decrease in NDE isolated from AD patients is similar to our CSF results. Furthermore, NDE NPTX2 correlated strongly with CSF biomarkers, p‐Tau_181_ and t‐Tau.

In this study, we present a novel, semi‐automated method for measuring NPTX2 in CSF using a very limited amount of sample volume. In CSF, our method strongly correlates with the quantification method currently used for IP‐MS, meaning that these two methods can be used interchangeably. In recent years, plasma biomarkers have risen to be the gold standard to strive for when developing a new assay due to their easy accessibility and lower cost, but more so because a blood sample is far less invasive for the patient compared to a lumbar puncture. Many assays have made the transition from CSF to blood^70^, ^71^, ^72^; however, this proves to be a challenge considering that plasma and serum have a much higher concentration of proteins,^73^, ^74^ leading to a greater protein matrix effect than that of CSF,^75^ which was also seen in this study. Yet, the measurement of NPTX2 in NDE seems a promising avenue for measuring synaptic pathology from blood samples.

## CONFLICT OF INTEREST STATEMENT

H.Z. has served at scientific advisory boards and/or as a consultant for Abbvie, Acumen, Alector, Alzinova, ALZPath, Amylyx, Annexon, Apellis, Artery Therapeutics, AZTherapies, Cognito Therapeutics, CogRx, Denali, Eisai, LabCorp, Merry Life, Nervgen, Novo Nordisk, Optoceutics, Passage Bio, Pinteon Therapeutics, Prothena, Red Abbey Labs, reMYND, Roche, Samumed, Siemens Healthineers, Triplet Therapeutics, and Wave, has given lectures sponsored by Alzecure, BioArctic, Biogen, Cellectricon, Fujirebio, Lilly, Novo Nordisk, Roche, and WebMD, and is a co‐founder of Brain Biomarker Solutions in Gothenburg AB (BBS), which is a part of the GU Ventures Incubator Program (outside submitted work). K.B. has served as a consultant and on advisory boards for Abbvie, AC Immune, ALZPath, AriBio, BioArctic, Biogen, Eisai, Lilly, Moleac Pte. Ltd., Neurimmune, Novartis, Ono Pharma, Prothena, Roche Diagnostics, Sanofi and Siemens Healthineers; has served at data monitoring committees for Julius Clinical and Novartis; has given lectures, produced educational materials and participated in educational programs for AC Immune, Biogen, Celdara Medical, Eisai and Roche Diagnostics; and is a co‐founder of Brain Biomarker Solutions in Gothenburg AB (BBS), which is a part of the GU Ventures Incubator Program, outside the work presented in this paper. D.A. participated in advisory boards from Fujirebio‐Europe, Roche Diagnostics, Grifols S.A., and Lilly, and received speaker honoraria from Fujirebio‐Europe, Roche Diagnostics, Nutricia, Krka Farmacéutica S.L., Zambon S.A.U., Neuraxpharm and Esteve Pharmaceuticals S.A. D.A. declares a filed patent application (WO2019175379 A1 Markers of synaptopathy in neurodegenerative disease). G.D. is the founder of LiBiCo LLC, which has no influence or contribution to the work presented in this manuscript. Author disclosures are available in the Supporting Information.

## CONSENT STATEMENT

All participants or their legally authorized representatives provided informed consent prior to any study‐related procedures.

## Supporting information



Supporting Information

Supporting Information
