# Supplementary material for: Development and validation of a novel Simoa assay for NPTX2 in Alzheimer's disease and Down syndrome
Source: Alzheimers Dement. 2025 Jun 16;21(6):e70241. doi: 10.1002/alz.70241 (PMC12168238; doi:10.1002/alz.70241)
Supplement: Supplementary file 2 — Supporting Information [file ALZ-21-e70241-s002.docx]

**Supplementary Table 1.** Demographics of the Discovery cohort.

| Discovery cohort (n=47) | CU  (n=23) | AD  (n=24) | *P-*value |
| --- | --- | --- | --- |
| Age, years | 70.2 (10.5, 42-85) | 76.8 (6.17, 66-87) | 0.03 |
| Females (%) | 7 (30.4) | 13 (54.2) | ns |
| INNOTEST CSF (pg/mL) |  |  |  |
| Aβ42 | 867 (286, 649-1030) | 488 (54.1, 437-518) | ˂0.001 |
| p-tau_181_ | 43 (4.45, 38-46) | 90.5 (30.4, 71.8-147) | ˂0.001 |
| t-tau | 250 (54.9, 213-344) | 846 (189, 532-946) | ˂0.001 |
| NFL | 1240 (652, 910-1740) | 2010 (904, 1405-2560) | 0.03 |

Notes: Notes: Data is presented as mean (standard deviation, min-max) for Age. For Aβ42, p-tau_181_, t-tau and NFL, data is presented as median (median absolute deviation, interquartile range). The Kruskal Wallis test was used to compare age and Pearson’s chi-square test was used to compare sex frequencies between groups. Biomarkers were compared using Mann-Whitney U test.

Abbreviations: Aβ42, β-amyloid 42; AD, Alzheimer’s disease; CSF, cerebrospinal fluid; CU, cognitively unimpaired; ns, non-significant; p-tau181, tau phosphorylated at threonine 181; t-tau, total tau.

**Supplementary Table 2:** Demographics of patients used in the western blot analysis.

| Brain ID | Sex | Diagnosis | Age | Neuropathological diagnosis (Braak) | PMD | APOE |
| --- | --- | --- | --- | --- | --- | --- |
| 3 | f | HC | 61 | 0 | 06:50 | 3/2 |
| 8 | f | HC | 64 | 0 | 08:35 | 2/4 |
| 19 | m | HC | 70 | 0 | 07:45 | 3/4 |
| 2 | f | AD | 61 | 6 | 06:25 | 4/4 |
| 10 | f | AD | 65 | 6 | 05:40 | 3/3 |
| 16 | m | AD | 69 | 6 | 05:30 | 3/3 |

**Validation of NPTX2 on MS**

To reliably correlate our Simoa method with the MS method, we validated the method with experiments to determine LLOQ, LOD and to test the parallelism. For LLOQ and LOD, the average concentration (attomole/µl) of 3 blanks (mean concentration = 0.48, Std = 0.06) plus 3 Std’s (LOD = 0.66 attomole/µl) and 10 Std’s (LLOQ = 1.08 attomole/µl) were calculated respectively (Supplementary Table 2). We also performed a parallelism experiment (Supplementary Fig. 3; Supplementary Table 11), where three different CSF samples were diluted down 5x and samples with signal below LLOQ were removed before calculating CV (%). The assay showed good parallelism for dilutions up to 1.67x, after that the % of expected concentrations drops significantly.


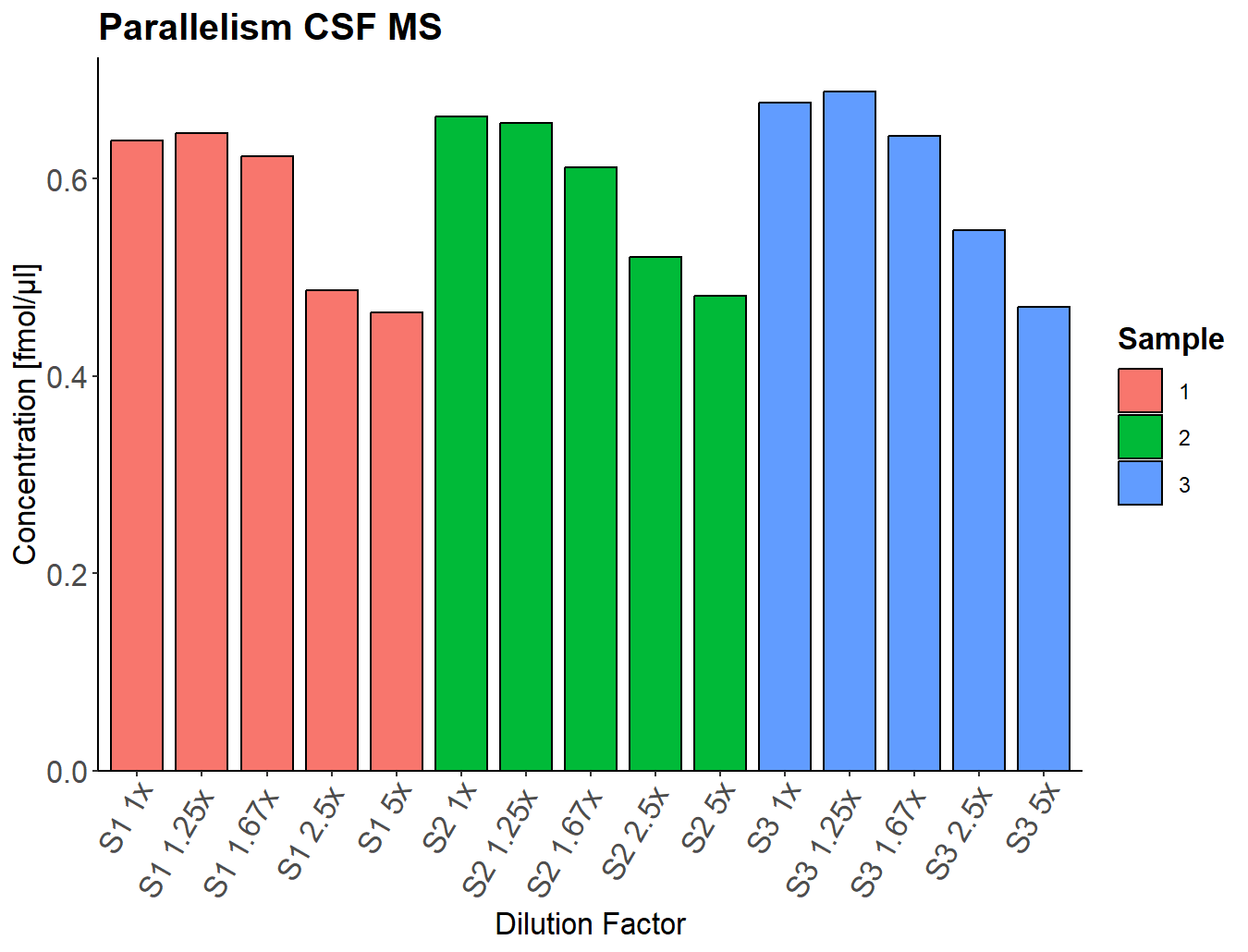


Supplementary Figure 1. CSF parallelism on the mass spectrometer.

Supplementary Table 3. Parallelism on the mass spectrometer.

|  | **Dilution** | **Calc. Conc (fmol/µL)** | **Measured Conc. (fmol/µL)** | **Expected Conc (fmol/µL)** | **Corrected dilution (fmol/µL)** | **% of Expected Conc** | **CV (%)** |
| --- | --- | --- | --- | --- | --- | --- | --- |
| **CSF 1** | 1,25 | 0,511 | 0,517 | 0,639 | 0,646 | 101 | 14.3 |
|  | 1,67 | 0,383 | 0,373 |  | 0,623 | 97 |  |
|  | 2,5 | 0,256 | 0,195 |  | 0,487 | 76 |  |
|  | 5 | 0,051 | 0,093 |  | 0,464 | 73 |  |
| **CSF 2** | 1,25 | 0,531 | 0,525 | 0,663 | 0,656 | 99 | 12.6 |
|  | 1,67 | 0,397 | 0,366 |  | 0,611 | 92 |  |
|  | 2,5 | 0,265 | 0,208 |  | 0,520 | 78 |  |
|  | 5 | 0,053 | 0,096 |  | 0,481 | 72 |  |
| **CSF 3** | 1,25 | 0,542 | 0,551 | 0,677 | 0,688 | 102 | 14.7 |
|  | 1,67 | 0,405 | 0,385 |  | 0,643 | 95 |  |
|  | 2,5 | 0,271 | 0,219 |  | 0,548 | 81 |  |
|  | 5 | 0,054 | 0,094 |  | 0,470 | 69 |  |

**Validation of NPTX2 on Simoa**

To validate the novel NPTX2 assay on the Simoa platform for CSF use, the following validation experiments were performed; lower limit of quantification (LLOQ), lower limit of detection (LOD), dilution linearity, parallelism, spike recovery and freeze thaw test. For LLOQ and LOD, the average concentration of 16 blanks (mean = 0.473 pg/mL, Std = 0.222 pg/mL) plus 3 Std’s (LOD = 1.14 pg/mL) and 10 Std’s (LLOQ = 2.70 pg/mL) was calculated respectively. The NPTX2 concentration was then interpolated from the calibration curve and multiplied by the sample dilution factor of 24x. For dilution linearity (Supplementary Fig.1A; Supplementary Table 3), three samples were spiked with recombinant NPTX2 (62.5 pg/mL) and then subsequently diluted down (2-fold, 4-fold, 8-fold, 16-fold, 32-fold) using sample diluent. The recovery rate for the dilution linearity test was calculated as shown below Supplementary Table 3. For parallelism (Supplementary Fig. 1B; Supplementary Table 4), the CSF samples were diluted as in the dilution linearity test and then the parallelism was subsequently calculated after samples with a signal lower than LLOQ were excluded. Spike recovery (Supplementary Fig. 1C; Supplementary Table 5) was performed by analysing three CSF samples, either untreated, or spiked with either 3.91 pg/mL, 15.63 pg/mL or 62.5 pg/mL recombinant NPTX2. The recovery rate for the Spike recovery test was calculated as shown below Supplementary Table 5. For the freeze-thaw stability test (Supplementary Fig. 1D; Supplementary Table 6), 3 samples were aliquoted and then underwent up to 5 freeze-thaw cycles before all being analysed together. Plasma was validated in the same manner as for CSF described above. Dilution linearity is shown in Supplementary Fig. 2A; Supplementary Table 7, parallelism in Supplementary Fig. 2B; Supplementary Table 8, spike recovery in Supplementary Fig. 2C; Supplementary Table 9 and freeze-thaw stability in Supplementary Fig 2D; Supplementary Table 10. An additional validation test was performed in plasma, a matrix effect test (Supplementary Fig. 2E). This was performed by diluting a plasma sample with a known concentration 2-fold down to 128x dilution and then all dilutions were spiked with the same concentration of recombinant NPTX2.





Supplementary Figure 2. CSF validation. (A) Linear dilution test. (B) Parallelism test. (C) Spike recovery test. (D) Freeze-thaw stability test.

Supplementary Table 4. Lower limit of quantification (Simoa and Mass spectrometry assay validation). LOD was calculated by adding 3 std’s to the mean concentration of 16 blanks. LLOQ was calculated by adding 10 std’s to the mean concentration of 16 blanks.

|  | SIMOA | Mass Spec |
| --- | --- | --- |
| Mean of blanks | 0.00382^1^ | 0.48^2^ |
| Std^3^ | 0.0005^1^ | 0.06^2^ |
| LOD^4^ (AEB) | 0.00518 |  |
| LLOQ^5^ (AEB) | 0.00836 |  |
| LOD | 0.274^6^ | 0.66^2^ |
| LLOQ | 0.647^6^ | 1.08^2^ |

^1^AEB; ^2^attomol/µL, ^3^Standard deviation; ^4^Lower limit of detection; ^5^Lower limit of quantification, ^6^pg/mL.

Supplementary Table 5. Dilution linearity (Simoa CSF assay validation). % Recovery was calculated as shown below the table.

|  | **Dilution** | **Measured Conc. (pg/mL)** | **Adjusted Conc (pg/mL)** | **Neat Sample (pg/mL)** | **Amount Spiked (pg/mL)** | **% Recovery** | **CV (%)** |
| --- | --- | --- | --- | --- | --- | --- | --- |
| **CSF 1** | 2 | 39,5 | 78,9 | 14,0 | 62,5 | 103 | 2,2 |
|  | 4 | 20,0 | 80,0 |  |  | 105 |  |
|  | 8 | 9,7 | 77,4 |  |  | 101 |  |
|  | 16 | 5,1 | 82,2 |  |  | 108 |  |
|  | 32 | 2,5 | 79,9 |  |  | 104 |  |
| **CSF 2** | 2 | 39,9 | 79,9 | 18,6 | 62,5 | 98 | 6,5 |
|  | 4 | 19,8 | 79,1 |  |  | 98 |  |
|  | 8 | 10,1 | 80,6 |  |  | 99 |  |
|  | 16 | 5,7 | 91,8 |  |  | 113 |  |
|  | 32 | 2,7 | 86,2 |  |  | 106 |  |
| **CSF 3** | 2 | 42,4 | 84,9 | 11,3 | 62,5 | 115 | 4,6 |
|  | 4 | 21,4 | 85,7 |  |  | 116 |  |
|  | 8 | 9,8 | 78,0 |  |  | 106 |  |
|  | 16 | 5,0 | 80,2 |  |  | 109 |  |
|  | 32 | 2,7 | 86,9 |  |  | 118 |  |
| **Buffer** | 2 | 31,6 | 63,3 | 0,1 | 62,5 | 101 | 5,9 |
|  | 4 | 17,5 | 69,9 |  |  | 112 |  |
|  | 8 | 7,9 | 63,1 |  |  | 101 |  |
|  | 16 | 3,8 | 60,8 |  |  | 97 |  |
|  | 32 | 1,9 | 60,6 |  |  | 97 |  |


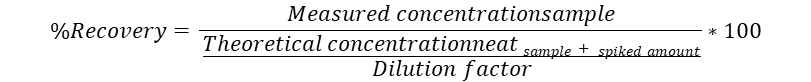


Supplementary Table 6. Parallelism (Simoa CSF assay validation).

|  | **Dilution** | **Calc. Conc (pg/mL)** | **Measured Conc. (pg/mL)** | **Expected Conc (pg/mL)** | **Corrected dilution (pg/mL)** | **% of Expected Conc** | **CV (%)** |
| --- | --- | --- | --- | --- | --- | --- | --- |
| **CSF 1** | 2 | 113,4 | 119,2 | 226,7 | 238,4 | 105 | 11.9 |
|  | 4 | 56,7 | 58,7 |  | 234,7 | 104 |  |
|  | 8 | 28,3 | 23,4 |  | 187,2 | 83 |  |
|  | 16 | 14,2 | N/A |  | N/A | N/A | <LLOQ^1^ |
|  | 32 | 7,1 | N/A |  | N/A | N/A | <LLOQ |
| **CSF 2** | 2 | 116,5 | 115,5 | 232,9 | 231,1 | 99 | 5.0 |
|  | 4 | 58,2 | 64,7 |  | 258,7 | 111 |  |
|  | 8 | 29,1 | 30,3 |  | 242,1 | 104 |  |
|  | 16 | 14,6 | N/A |  | N/A | N/A | <LLOQ |
|  | 32 | 7,3 | N/A |  | N/A | N/A | <LLOQ |
| **CSF 3** | 2 | 232,1 | 217,5 | 464,2 | 435,1 | 94 | 5.6 |
|  | 4 | 116,1 | 115,2 |  | 460,9 | 99 |  |
|  | 8 | 58,0 | 55,5 |  | 444,2 | 96 |  |
|  | 16 | 29,0 | 31,9 |  | 510,7 | 110 |  |
|  | 32 | 14,5 | N/A |  | N/A | N/A | <LLOQ |

^1^Lower limit of quantification.

Supplementary Table 7. Spike recovery (Simoa CSF assay validation). % Recovery was calculated as shown below the table.

| **Sample** | **Spike conc. (pg/mL)** | **Non-spiked conc (pg/mL)** | **Expected (pg/mL)** | **Measured Conc (pg/mL)** | **Recovery (%)** |
| --- | --- | --- | --- | --- | --- |
| **CSF 1** | 3,91 | 14,0 | 17,9 | 19,2 | 134,4 |
|  | 15,63 |  | 29,6 | 28,4 | 92,0 |
|  | 62,5 |  | 76,5 | 77,1 | 100,9 |
| **CSF 2** | 3,91 | 18,6 | 22,5 | 21,4 | 72,2 |
|  | 15,63 |  | 34,2 | 30,5 | 76,5 |
|  | 62,5 |  | 81,1 | 74,6 | 89,6 |
| **CSF 3** | 3,91 | 11,3 | 15,2 | 14,2 | 73,6 |
|  | 15,63 |  | 26,9 | 27,5 | 103,9 |
|  | 62,5 |  | 73,8 | 70,7 | 95,1 |


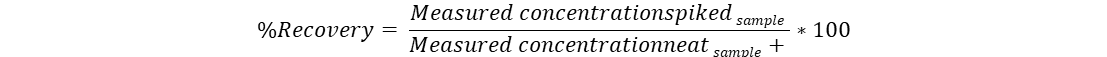


Supplementary Table 8. Freeze-Thaw cycles (Simoa CSF validation).

| **Sample** | **Freeze-Thaw Cycle** | **Measured Conc. (pg/mL)** | **Normalized Conc. (%)** | **CV (%)** |
| --- | --- | --- | --- | --- |
| **CSF 1** | 0 | 95,9 | 100 | 21,3 |
|  | 1 | 93,4 | 97,5 |  |
|  | 3 | 62,2 | 64,8 |  |
|  | 5 | 69,0 | 72,0 |  |
| **CSF 2** | 0 | 499,4 | 100 | 5,7 |
|  | 1 | 450,9 | 90,3 |  |
|  | 3 | 501,7 | 100,5 |  |
|  | 5 | 456,5 | 91 |  |
| **CSF 3** | 0 | 946,0 | 100 | 4,9 |
|  | 1 | 860,3 | 90,9 |  |
|  | 3 | 961,9 | 101,7 |  |
|  | 5 | 933,9 | 98,7 |  |





Supplementary Figure 3. Plasma validation. (A) Linear dilution test. (B) Parallelism test. (C) Spike recovery test. (D) Freeze-thaw test. (E) Matrix effect test.

Supplementary Table 9. Dilution linearity (Simoa plasma assay validation).

|  | **Dilution** | **Measured Conc. (pg/mL)** | **Adjusted Conc (pg/mL)** | **Neat Sample (pg/mL)** | **Amount Spiked (pg/mL)** | **% Recovery** | **CV (%)** |
| --- | --- | --- | --- | --- | --- | --- | --- |
| **Plasma 1** | 2 | 4,5 | 9,0 | 8,0 | 31,3 | 23 | 31,2 |
|  | 4 | 2,2 | 8,9 |  |  | 23 |  |
|  | 8 | 1,1 | 9,0 |  |  | 23 |  |
|  | 16 | 0,6 | 9,6 |  |  | 25 |  |
|  | 32 | 0,5 | 16,5 |  |  | 42 |  |
| **Plasma 2** | 2 | 8,9 | 17,8 | 18,6 | 31,3 | 36 | 18,0 |
|  | 4 | 4,4 | 17,5 |  |  | 35 |  |
|  | 8 | 2,5 | 19,6 |  |  | 40 |  |
|  | 16 | 1,4 | 21,9 |  |  | 44 |  |
|  | 32 | 0,8 | 26,5 |  |  | 54 |  |
| **Plasma 3** | 2 | 18,4 | 36,8 | 31,4 | 31,3 | 59 | 14,4 |
|  | 4 | 8,8 | 35,3 |  |  | 56 |  |
|  | 8 | 4,4 | 35,3 |  |  | 56 |  |
|  | 16 | 2,5 | 40,2 |  |  | 64 |  |
|  | 32 | 1,5 | 48,7 |  |  | 78 |  |
| **Buffer** | 2 | 14,7 | 29,3 | 0,1 | 31,3 | 93 | 3,4 |
|  | 4 | 7,7 | 30,8 |  |  | 98 |  |
|  | 8 | 3,7 | 29,2 |  |  | 93 |  |
|  | 16 | 2,0 | 31,5 |  |  | 101 |  |
|  | 32 | 0,9 | 29,6 |  |  | 94 |  |

% Recovery was calculated as described in the CSF validation.

Supplementary Table 10. Parallelism (Simoa plasma assay validation).

|  | **Dilution** | **Calc. Conc (pg/mL)** | **Measured Conc. (pg/mL)** | **Expected Conc (pg/mL)** | **Corrected dilution (pg/mL)** | **% of Expected Conc** | **CV (%)** |
| --- | --- | --- | --- | --- | --- | --- | --- |
| **Plasma 1** | 2 | 4,0 | 4,6 | 8,0 | 9,3 | 116 | 13.2 |
|  | 4 | 2,0 | 1,9 |  | 7,7 | 97 |  |
|  | 8 | 1,0 | 0,9 |  | 7,4 | 93 |  |
|  | 16 | 0,5 | 0,6 |  | 9,7 | 122 |  |
|  | 32 | 0,5 | N/A |  | N/A | N/A | <LLOQ^1^ |
| **Plasma 2** | 2 | 9,1 | 8,8 | 18,1 | 17,7 | 97 | 25.3 |
|  | 4 | 4,5 | 4,2 |  | 16,7 | 92 |  |
|  | 8 | 2,3 | 2,3 |  | 18,3 | 101 |  |
|  | 16 | 1,1 | 1,3 |  | 20,3 | 112 |  |
|  | 32 | 0,6 | 0,9 |  | 29,5 | 162 |  |
| **Plasma 3** | 2 | 15,7 | 15,6 | 31,4 | 31,2 | 99 | 13.2 |
|  | 4 | 7,9 | 7,4 |  | 29,8 | 95 |  |
|  | 8 | 3,9 | 3,8 |  | 30,0 | 96 |  |
|  | 16 | 2,0 | 2,3 |  | 36,0 | 115 |  |
|  | 32 | 1,0 | 1,2 |  | 39,9 | 127 |  |

^1^Lower limit of quantification.

Supplementary Table 11. Spike recovery (Simoa plasma assay validation).

| Sample | Spike conc. (pg/mL) | Non-spiked conc (pg/mL) | Expected (pg/mL) | Measured Conc (pg/mL | Recovery (%) |
| --- | --- | --- | --- | --- | --- |
| Plasma 1 | 7,81 | 8,0 | 15,8 | 8,1 | 1,2 |
|  | 31,2 |  | 39,2 | 9,0 | 3,2 |
|  | 125 |  | 133,0 | 9,9 | 1,5 |
| Plasma 2 | 7,81 | 18,6 | 26,4 | 17,4 | -14,8 |
|  | 31,2 |  | 49,8 | 17,0 | -5,1 |
|  | 125 |  | 143,6 | 19,0 | 0,3 |
| Plasma 3 | 7,81 | 31,4 | 39,2 | 34,6 | 40,4 |
|  | 31,2 |  | 62,6 | 34,2 | 9,1 |
|  | 125 |  | 156,4 | 33,1 | 1,3 |

% Recovery was calculated as described in the CSF validation.

Supplementary Table 12. Freeze-Thaw cycles (Simoa CSF validation).

| Sample | Freeze Thaw Cycle | Measured Conc. (pg/mL) | Normalized Conc. (%) | CV (%) |
| --- | --- | --- | --- | --- |
| Plasma 1 | 0 | 11,1 | 100 | 4,4 |
|  | 1 | 10,0 | 90,1 |  |
|  | 3 | 10,8 | 97,6 |  |
|  | 5 | 10,6 | 95,4 |  |
| Plasma 2 | 0 | 11,6 | 100 | 9,5 |
|  | 1 | 9,3 | 80,0 |  |
|  | 3 | 11,0 | 95,1 |  |
|  | 5 | 11,2 | 96 |  |
| Plasma 3 | 0 | 8,8 | 100 | 8,5 |
|  | 1 | 8,0 | 91,4 |  |
|  | 3 | 8,2 | 93,2 |  |
|  | 5 | 7,1 | 81,1 |  |


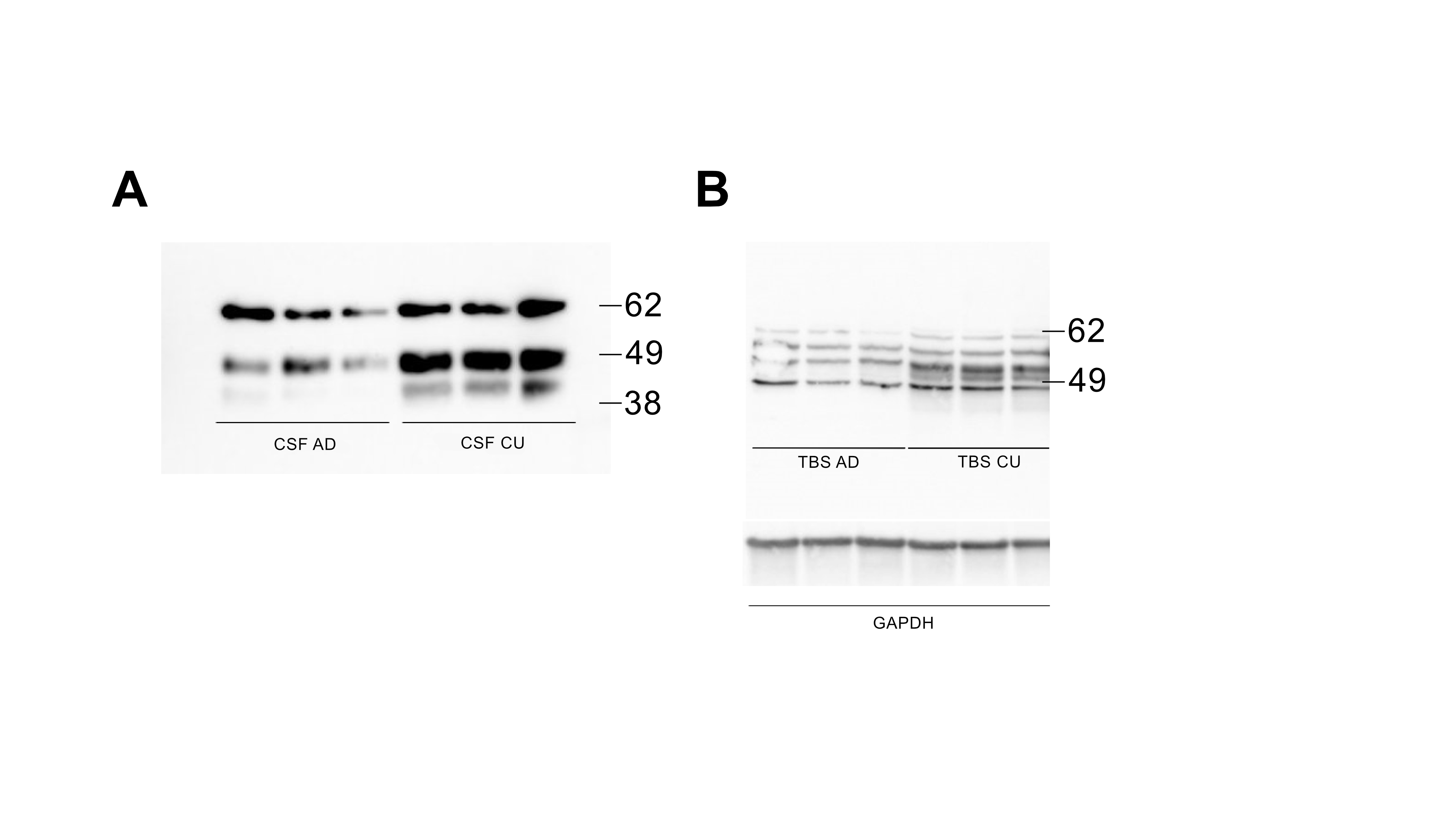


**Supplementary Figure 4.** Western blot for NPTX2 in (**A**) CSF CU (n = 3) and AD (n = 3) 1.19 µg/sample protein loaded, (**B**) TBS CU (n = 3) and AD (n = 3) 10 µg/sample protein loaded.



Supplementary Figure 5. Correlations for biomarkers using Spearman Rank correlation. BioFINDER pilot cohort, (A) all patients (n = 96), (B) CU group only (n = 49), (C) AD group only (n =47). DABNI cohort, (D) all patients (n = 188), (E) CU group only (n = 17), (F) DS groups combined (n =171), (G) pDS group only (n = 19), (H) aDS group only (n = 73), (I) dDS group only (n = 79). NDE, (G) all patients (n = 45), (H) CU group only (n = 22), (I) DS group only (n = 23). P-values are indicated by asterisks, * = p < 0.05, ** = p < 0.01 and *** = p < 0.001.


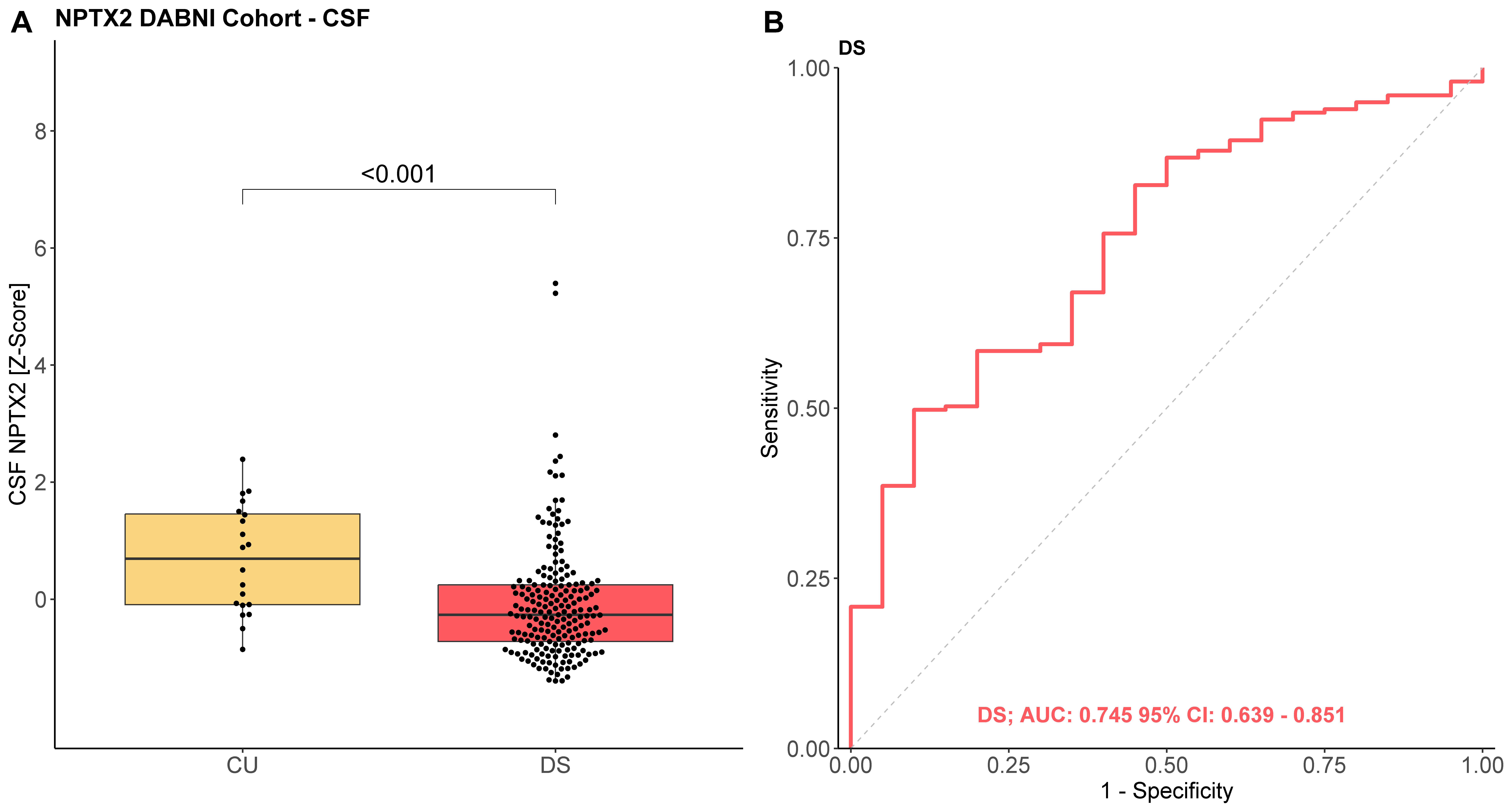


**Supplementary Figure 6.** CSF NPTX2 in the DABNI cohort. (**A**) Groupwise comparison between CU (n = 20) and DS (n = 197). (**B**) ROC curve for NPTX2 in DS compared to CU.





Supplementary Figure 7. Associations in BioFINDER pilot cohort. CSF pTau_181_ (log^2^ z-score) with MMSE score in (A) all patients (n = 95), (B) CU group (n = 49), (C) AD group (n =46). CSF pTau_181_ (z-score) with Tau PET in (D) all patients (n = 95), (E) CU group (n = 49), (F) AD group (n = 46). CSF pTau_181_ (z-score) with Amyloid PET in (G) all patients (n = 78), (H) CU group (n = 49), (I) AD group (n = 29). pTau_181_ (z-score) with cortical thickness in (J) all patients (n = 96), (K) CU group (n = 49), (L) AD group (n = 47). Log_2_ values of CSF pTau_181_ was used, with age and sex as covariates for the calculation of linear models.





Supplementary Figure 8. Associations in BioFINDER pilot cohort. CSF NFL (log^2^ z-score) with MMSE score in (A) all patients (n = 95), (B) CU group (n = 49), (C) AD group (n =46). CSF NFL (z-score) with Tau PET in (D) all patients (n = 95), (E) CU group (n = 49), (F) AD group (n = 46). CSF NFL (z-score) with Amyloid PET in (G) all patients (n = 78), (H) CU group (n = 49), (I) AD group (n = 29). CSF NFL (z-score) with cortical thickness in (J) all patients (n = 96), (K) CU group (n = 49), (L) AD group (n = 47). Log_2_ values of NFL was used, with age and sex as covariates for the calculation of linear models.





Supplementary Figure 9. Associations of CSF NPTX2, pTau and NFL in DS patients with CRT. CSF NPTX2 in (A) all DS individuals (n = 78), (B) aDS (n = 40), (C) pDS (n = 8) and (D) dDS (n = 30). CSF pTau_181_ in (E) all DS individuals (n = 78), (F) aDS (n = 40), (G) pDS (n = 8) and (H) dDS (n = 30). CSF NFL in (I) all DS individuals (n = 74), (J) aDS (n = 37), (K) pDS (n = 8) and (L) dDS (n = 29). Log_2_ values of CSF NPTX2, pTau_181_, and NFL was used, with age and sex as covariates for the calculation of linear models.





**Supplementary Figure 10.** Plasma NPTX2 in BioFINDER pilot cohort. (**A**) groupwise comparison between CU (n = 49) and AD (n = 48). Correlations between NPTX2 in CSF and plasma measured on the Simoa for all patients (**B**), CU group alone (**C**) and AD group alone (**D**).


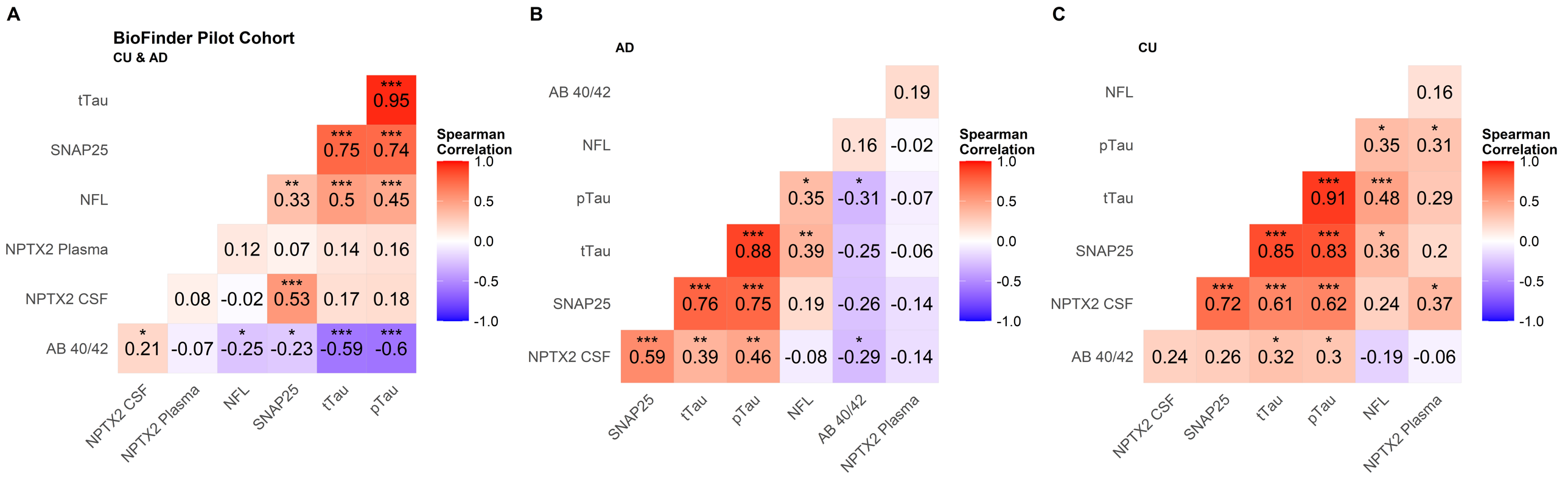


Supplementary Figure 11. Correlations with NPTX2 in plasma with other CSF biomarkers in BioFINDER pilot cohort. (A) All patients (n = 94), (B) AD patients only (n = 47), (C) CU patients only (n = 47). P-values are indicated by asterisks, * = p < 0.05, ** = p < 0.01 and *** = p < 0.001.





Supplementary Figure 12 Associations in BioFINDER pilot cohort. Plasma NPTX2 (z-score) with MMSE score in (A) all patients (n = 96), (B) CU group (n = 49), (C) AD group (n =47). Plasma NPTX2 (z-score) with Tau PET in (D) all patients (n = 96), (E) CU group (n = 49), (F) AD group (n = 47). Plasma NPTX2 (z-score) with Amyloid PET in (G) all patients (n = 78), (H) CU group (n = 49), (I) AD group (n = 29). Plasma NPTX2 (z-score) with cortical thickness in (J) all patients (n = 97), (K) CU group (n = 49), (L) AD group (n = 48). Log_2_ values of plasma NPTX2 was used, with age and sex as covariates for the calculation of linear models.
